# A systematic review and meta-analysis of adolescent nutrition in Ethiopia: Transforming adolescent lives through nutrition (TALENT) initiative

**DOI:** 10.1371/journal.pone.0280784

**Published:** 2023-04-06

**Authors:** Mubarek Abera, Abdulhalik Workicho, Melkamu Berhane, Desta Hiko, Rahma Ali, Beakal Zinab, Abraham Haileamlak, Caroline Fall

**Affiliations:** 1 Faculty of Medical Sciences, Institute of Health, Jimma University, Jimma, Ethiopia; 2 Faculty of Public Health, Institute of Health, Jimma University, Jimma, Ethiopia; 3 Medical Research Council (MRC) Lifecourse Epidemiology Centre, Southampton General Hospital, University of Southampton, Southampton, United Kingdom; Pokhara University, NEPAL

## Abstract

**Background:**

Ethiopia has undergone rapid economic growth over the last two decades that could influence the diets and nutrition of young people. This work systematically reviewed primary studies on adolescent nutrition from Ethiopia, to inform future interventions to guide policies and programs for this age group.

**Method:**

A systematic search of electronic databases for published studies on the prevalence of and interventions for adolescent malnutrition in Ethiopia in the English language since the year 2000 was performed using a three-step search strategy. The results were checked for quality using the Joanna Bridge Institute (JBI) checklist, and synthesized and presented as a narrative description.

**Results:**

Seventy six articles and two national surveys were reviewed. These documented nutritional status in terms of anthropometry, micronutrient status, dietary diversity, food-insecurity, and eating habits. In the meta-analysis the pooled prevalence of stunting, thinness and overweight/obesity was 22.4% (95% CI: 18.9, 25.9), 17.7% (95% CI: 14.6, 20.8) and 10.6% (7.9, 13.3), respectively. The prevalence of undernutrition ranged from 4% to 54% for stunting and from 5% to 29% for thinness. Overweight/obesity ranged from 1% to 17%. Prevalence of stunting and thinness were higher in boys and rural adolescents, whereas overweight/obesity was higher in girls and urban adolescents. The prevalence of anemia ranged from 9% to 33%. Approximately 40%-52% of adolescents have iodine deficiency and associated risk of goiter. Frequent micronutrient deficiencies are vitamin D (42%), zinc (38%), folate (15%), and vitamin A (6.3%).

**Conclusions:**

The adolescent population in Ethiopia is facing multiple micronutrient deficiencies and a double-burden of malnutrition, although undernutrition is predominant. The magnitude of nutritional problems varies by gender and setting. Context-relevant interventions are required to effectively improve the nutrition and health of adolescents in Ethiopia.

## Introduction

Adolescence, the transition from childhood to adulthood, is a period of rapid growth and development, second only to fetal life and infancy. About 20% and 40% of the final adult height and weight respectively are attained during this stage [1]. Physical growth (muscle and bone size and density), the onset of puberty and (in girls) menstruation, and major psychological, emotional and cognitive maturation, are vital changes taking place during adolescence, creating a greater demand for protein and energy [2], and a continued need for micronutrients [3]. Equally, the rapid mental, psychological and social development during adolescence increases the risk of psychosocial problems, [4] which may alter adolescents’ nutrition behavior.

A 2018 United Nation Children’s Fund (UNICEF) report showed that globally, adolescents constitute 16% of the population [5]. However, this figure in Ethiopia is 34% [6]. Traditionally, under nutrition has been the predominant public health nutrition problem in low income settings, while in high income countries overnutrition (overweight and obesity) was more prevalent. However, as many low- and middle-income countries (LMICs) are experiencing rapid economic growth, with concomitant urbanisation and lifestyle transformations, overweight and obesity are emerging as significant problems among the adolescent population. This, alongside persisting undernutrition in large sections of the population results in a double burden of malnutrition in LMICs [7], compounded by low levels of government investment to solve the problem [8, 9].

Malnutrition in LMICs often starts prenatally, continues through childhood and adolescence, and even extends to adulthood. This creates a vicious cycle of malnutrition contributing to adverse intergenerational effects, such as low birth weight, which in turn has lifelong effects on health [10]. Adolescence can be viewed as a period of opportunity in which catch-up growth can occur and when nutritional status can be optimized prior to parenthood [11]. Thus designing appropriate interventions to support safe, healthy and productive transition from childhood to adulthood is a critical step to end malnutrition and improve the overall health and wellbeing of society [12].

Although nutritional interventions are growing rapidly in Ethiopia, most of these are targeted to young children, and pregnant and lactating women, leaving adolescents a relatively neglected group. Many of the successes achieved in maternal and child health in Ethiopia over the last few decades are the result of specific investment in the health and nutrition of children and women. Though the 2017 revised National Nutrition Program (NNP) and the 2018 Food and Nutrition Policy (FNP) [13, 14] highlighted issues of adolescent nutrition, there is no equivalent investment in adolescents’ distinctive health and nutrition needs in Ethiopia [15]. In order to inform this process, we have conducted this systematic review and meta-analysis of original research from Ethiopia, thereby collating current evidence on nutritional status of Ethiopian adolescents and the effectiveness of nutritional interventions in this age group.

## Methods

### Search strategy

We adopted a rigorous systematic approach [16, 17]. The first step was to formulate objectives/research questions as follows.

1. From observational studies and surveys, what is known about the nutritional status of Ethiopian adolescents (boys and girls age 10–19 years) in terms of: a) body size and energy balance (chiefly weight, BMI and height), b) micronutrient status, c) dietary intake, diversity and quality, and d) dietary behaviours?
2. From their associations with population characteristics, what is known about the possible determinants of these aspects of nutritional status? Which adolescents are at risk of nutritional problems?
3. From intervention studies, what is known about the effectiveness of nutritional interventions in the Ethiopian population?

A three-step comprehensive literature search strategy was used to locate relevant literature published over the last 20 years from Ethiopia. Firstly, we set relevant key words and terms, using a logic grid for each key term. The terms used included “nutrition”, “micronutrient”, “malnutrition”, “undernutrition”, stunting”, “thin”, “obesity”, “food insecurity” “dietary diversity” “anemia”, "iron", "folic", "vitamin", "zinc", "iodine", "copper", "magnesium", "selenium" and “eating disorder”. The terms we used to define the population were “adolescent”, “teenage”, “youth”, “school children” and “young child”, and setting in “Ethiopia”.

### Data sources/base

The search query was first developed for PubMed and later extended to EBSCO/ERIC and EBSCO/CINAHL to identify different concepts in the literature. Secondly, we carried out the search, expanding all terms in specific databases. Thirdly, we manually searched the reference lists of the identified studies.

### Study selection process

Following the search, two researchers (AW & DH) screened studies by title. Then two independent researchers (BZ & RA) screened the abstracts and assessed the eligibility for full text retrieval. Selected full-text studies were compared between the reviewers, with disagreements being resolved through discussion and consensus with a 3^rd^ researcher (MA).

### Population

Adolescent age 10–19

### Outcomes

Nutritional status measured by anthropometric indices: Stunting (Height-for-age z-score <-2) Thinness (BMI-for-age z-score <-2), underweight (weight-for-age z-score <-2), overweight (Body mass index-for-age z-score is >1), Obesity (Body mass index-for-age z-score is >2), combined overweight/obesity (Body mass index-for-age z-score is >1) micronutrient status, dietary diversity score (DDS) measured with the Food and Agriculture Organization of the United Nations (FAO), and food insecurity score assessed with Household Food Insecurity Access Scale (HFIAS).

### Study selection criteria

#### Inclusion criteria

The inclusion criteria were developed through discussion in an iterative process. Primary studies or national government surveys (DHS and Micronutrient survey) involving human subjects, reporting quantitative outcomes, published in English between the year 2000 and 2020, conducted in Ethiopia, among adolescents aged 10–19 years were included. Therefore, in this review, studies which researched nutritional status including but not limited to under nutrition, over nutrition, micronutrient status or deficiency, food insecurity, diet diversity, dietary behavior, diet quality, eating disorders and protein energy deficiency among adolescents in Ethiopia were included. A PRISMA flow diagram is included to inform the study selection process.

#### Exclusion criteria

Unpublished studies, articles published in a language other than English, reviews, book reviews, commentaries, letters to the editor and case reports, publications with only an abstract, and studies conducted outside Ethiopia were excluded. Qualitative studies were excluded because these are reviewed elsewhere by the TALENT collaboration [18].

### Data charting and synthesis of the results

Data were extracted using a pre-tested two-step process. Firstly, we developed a template (authors, year, settings (urban/rural), methodology, study question, study design, population, outcomes, and study quality). Secondly, each reviewer independently extracted data, which was then compared and any discrepancies discussed and resolved. The study findings are synthesized using narrative descriptions based on individual indicators that emerged. Meta-analysis was done for stunting, thinness and overnutrition (overweight/obesity) using random effect model with restricted maximum likelihood (REML) method. We used STATA version 17 for the meta-analysis. Forest plot for proportion with 95% confidence interval (CI) was reported. A Preferred Reporting Items for Systematic reviews and Meta-Analyses Checklist was used to guide reporting [19].

### Quality assessment

The Joanna Briggs Institute (JBI) quality assessment checklists for observational [20] and interventional [21] studies were used and the quality of the reviewed studies rated as low, medium and high. Studies rated as low quality were excluded from the review.

## Results

### Study selection

**Fig 1:**
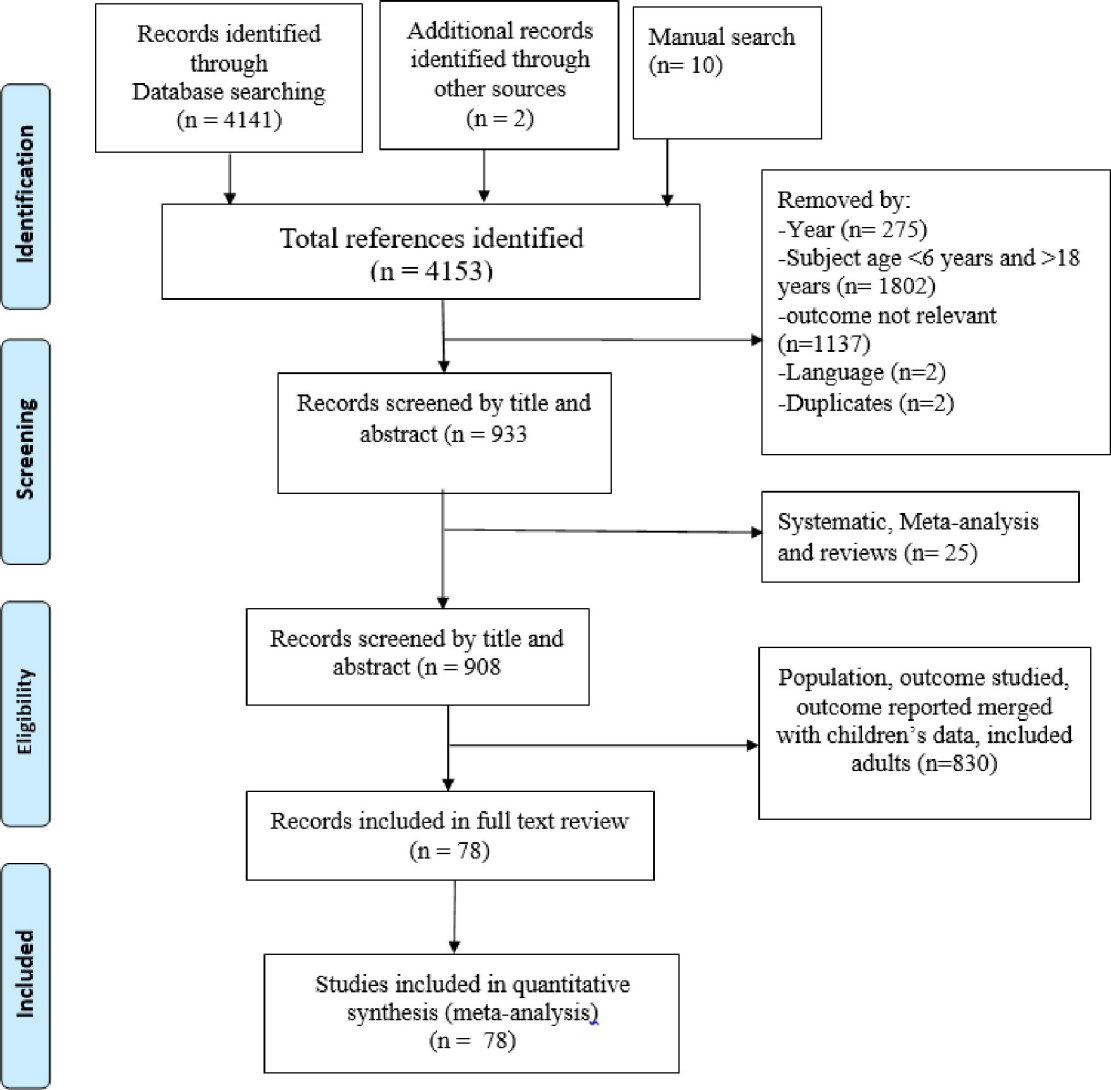
Flow chart for search and selection process of articles on adolescent nutrition and health in Ethiopia.

The initial search strategy and additional manual search identified 4153 records of which 3220 were removed due to the age of the study population (1810), outcomes mixed with other non-relevant indicators (1131), year of publication (275), language (2) and duplicates (2), leaving 933 records (Fig 1). Of these, 25 studies were secondary reviews or meta-analyses, which resulted in 908 records for full text review. Through full text review, 830 records were excluded as they were not relevant to our objectives, leaving 78 studies. Then finally a total of 74 articles were used for data extraction. No studies were excluded because of poor quality. Our last search date was on 1, November 2022.

### Characteristics of included studies

The majority of the reviewed studies were observational and cross sectional; there were six longitudinal [22–27] and one interventional (quasi experimental) [28] studies. The outcomes assessed included body size and growth (41 studies) [22, 28–66], micronutrient status and deficiencies (15 studies) [39, 67–80], diet diversity (9 studies) [28, 44, 72, 80–85] and food insecurity (7 studies) [23–27, 46, 86]. There were two studies which addressed eating disorders [87, 88]. Ten studies reported multiple outcomes, and are described in more than one section of the review.

### Findings of the review

#### Nutritional status defined by anthropometry, and its determinants

Forty studies, on a sample of 25 397 adolescents, and one demographic and health survey (DHS) [75] addressed nutritional status as defined by height, weight and BMI. Five specifically assessed over-nutrition (overweight and/or obesity) [43, 44, 48, 50, 53], Eight assessed both undernutrition (stunting, thinness or underweight) and over-nutrition [29, 31, 32, 39–41, 45, 89] and 25 reported only undernutrition [22, 30, 33–38, 42, 46, 47, 49, 51, 52, 55–66]. The national adolescent nutrition surveys (DHS) reported stunting, thinness and overweight. Twenty one (including the DHS) were conducted in mixed urban and rural settings while thirteen and six studies were conducted only in urban and rural settings respectively. Two did not describe the study setting.

From studies that reported Z-scores for adolescents’ height-for-age (HAZ) and body-mass-index (BMI)-for-age (BAZ) using the 2007 WHO growth reference, the minimum and maximum mean HAZ was -1.5 [33] and -0.5 [41] while they were -1.29 [33] and 0.44 [89] for BAZ respectively. A comparison between urban and rural adolescents showed that the mean BAZ and HAZ were significantly higher in urban than rural adolescents, with mean differences of 0.2 (95% confidence interval (CI): 0.02–0.34) and 0.58 (95% CI 0.45–0.72), respectively [32]. Both HAZ and BAZ, even for urban adolescents were, however, lower than the WHO reference data [32].

**Fig 2:**
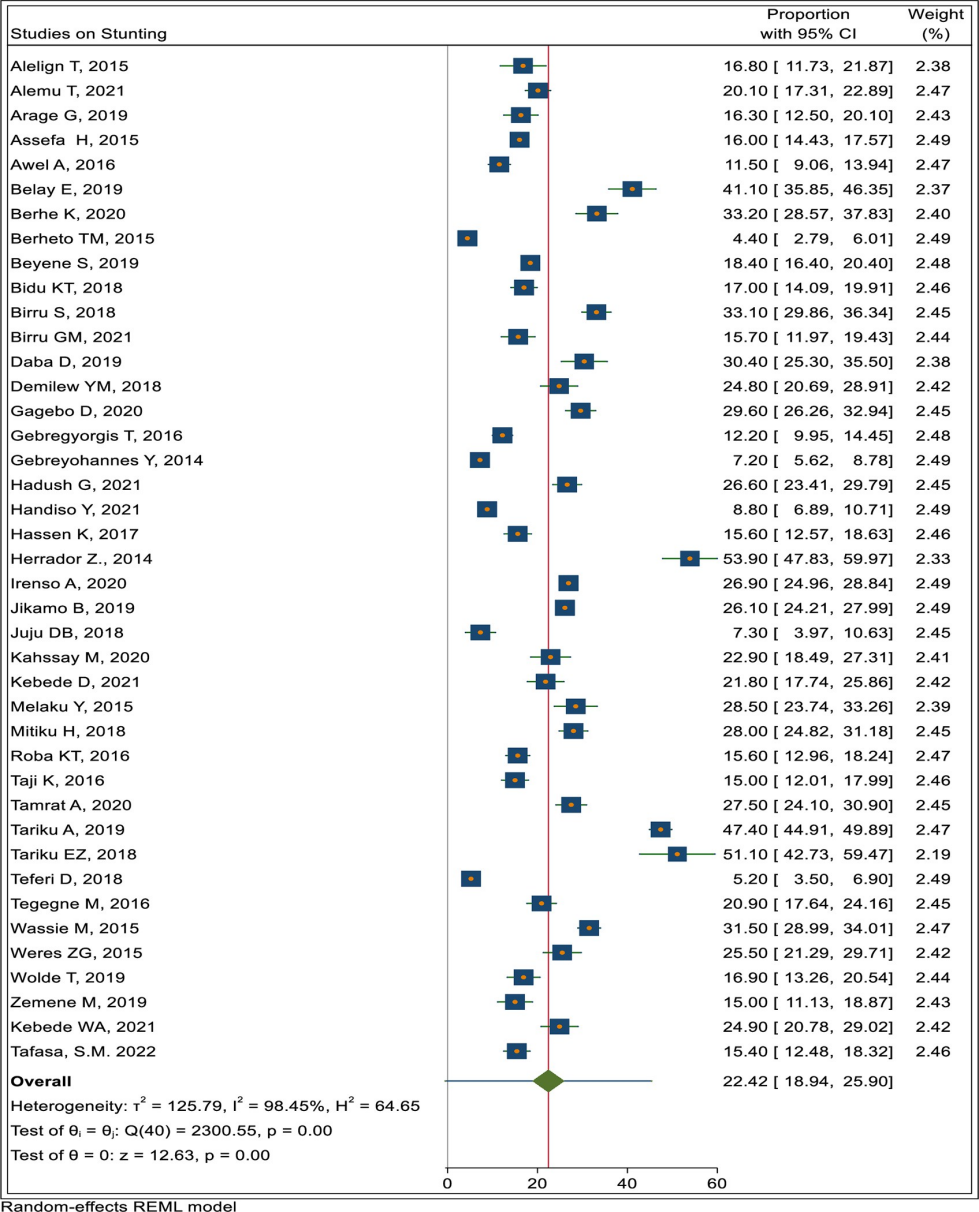
Pooled prevalence of adolescent stunting in Ethiopia.

**Fig 3:**
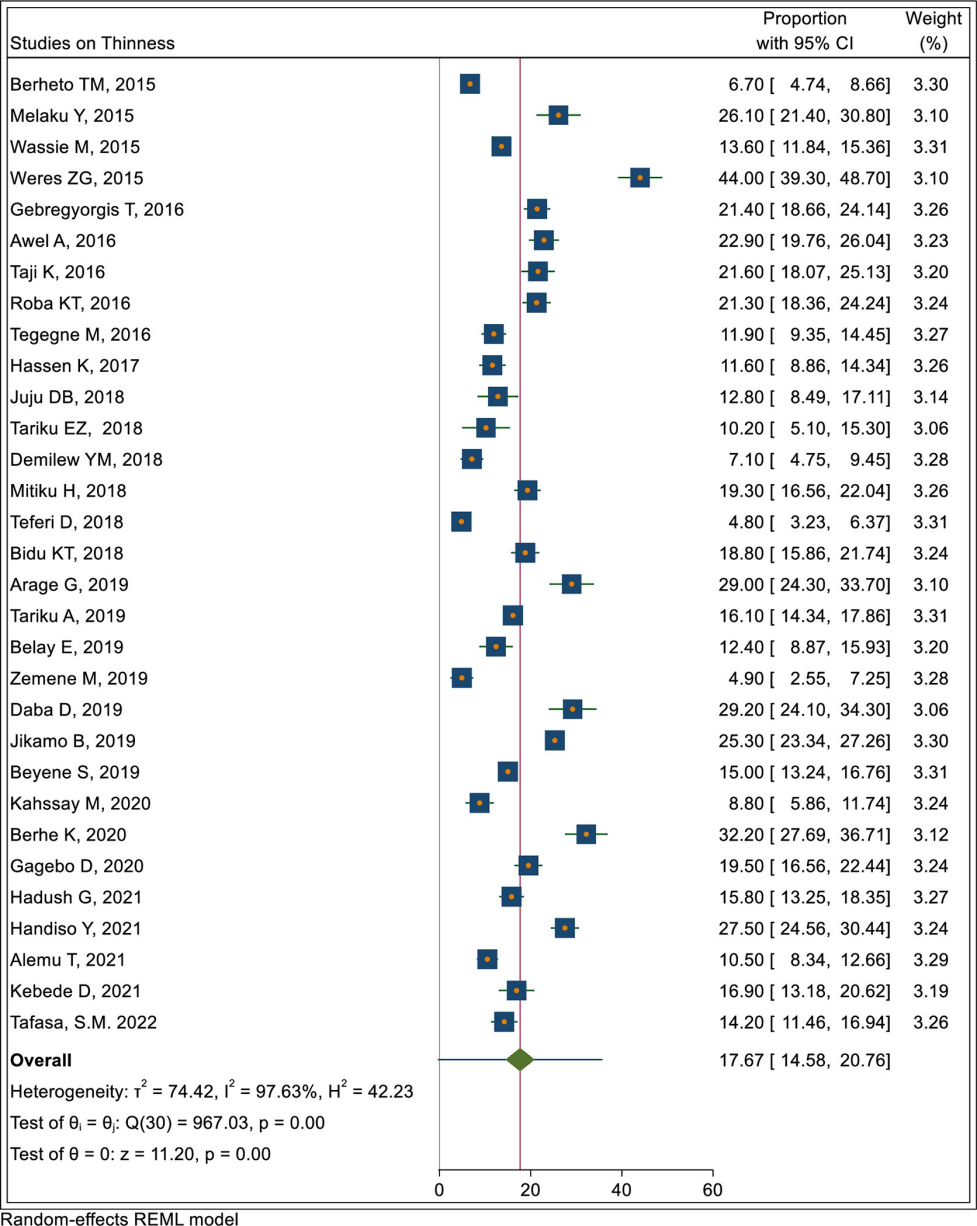
Pooled prevalence of adolescent thinness in Ethiopia.

**Fig 4:**
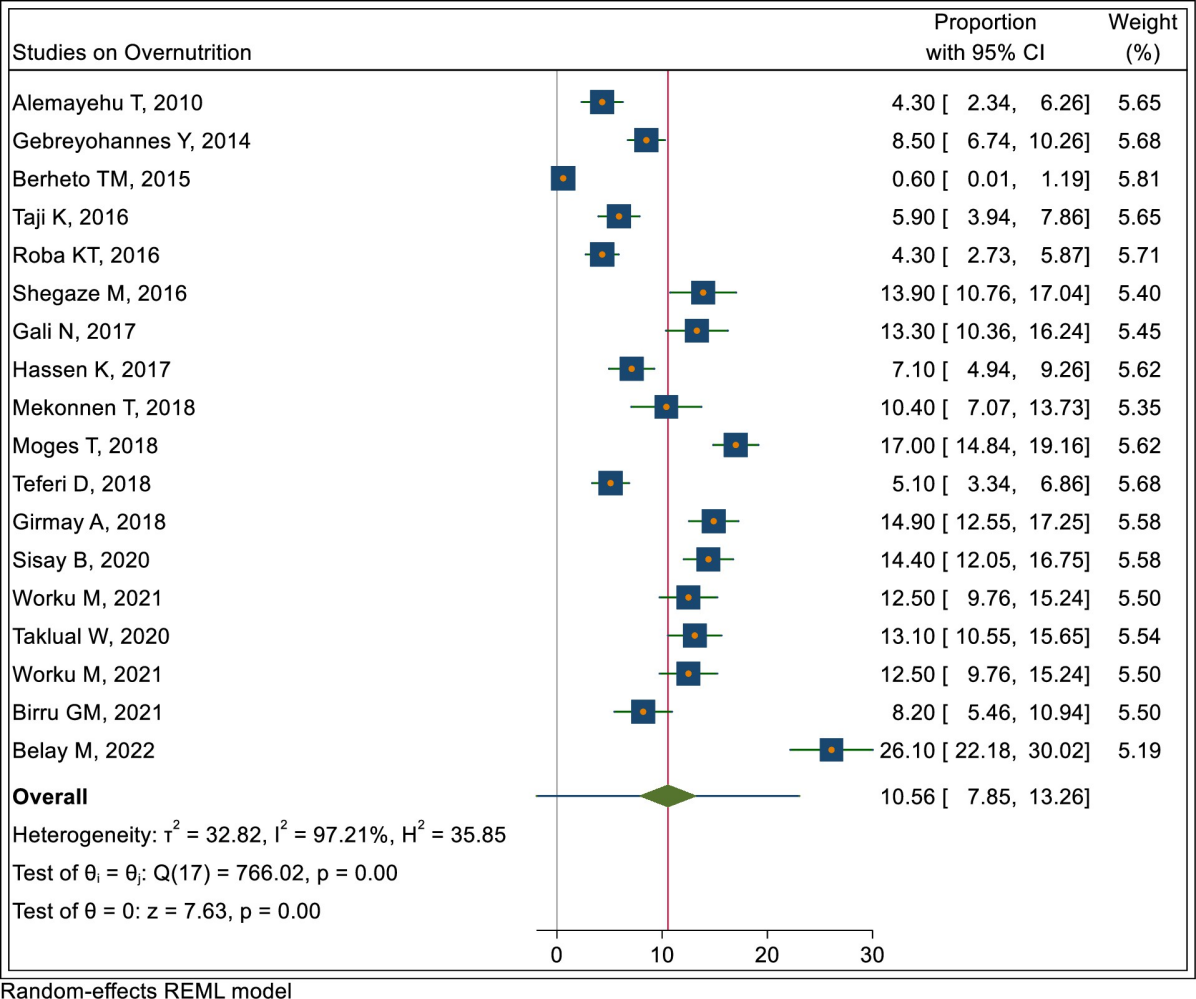
Pooled prevalence of overnutrition (overweight/obesity) in Ethiopia.

In the meta-analysis the pooled prevalence of stunting, thinness and overnutrition (overweight/obesity) were 22.4% (95% CI: 18.9, 25.9), 17.7% (95% CI: 14.6, 20.8) and 10.6% (7.9, 13.3), respectively as shown in Figs 2–4.

#### Nutritional status by sex and setting

**Fig 5:**
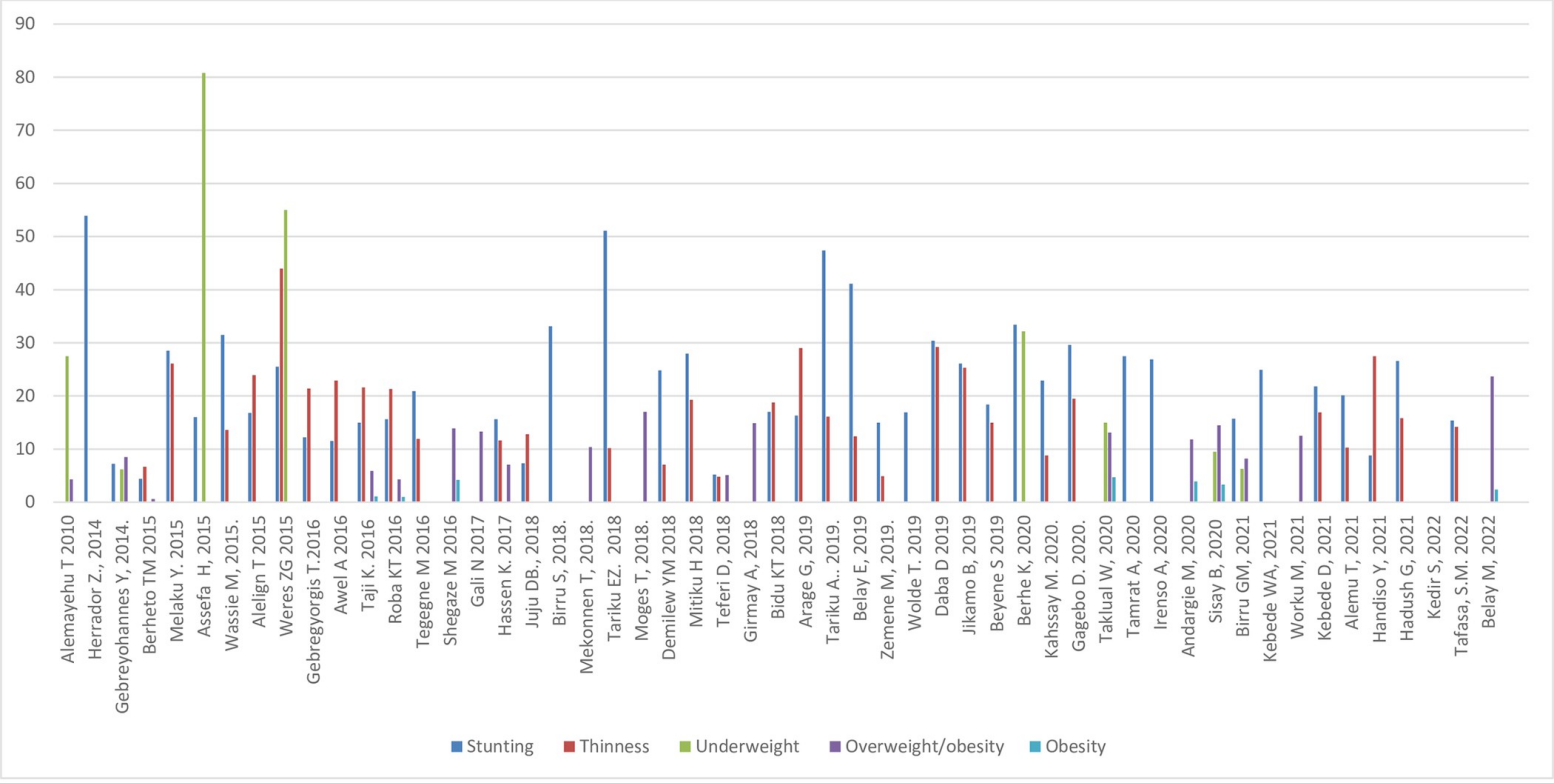
Trends in the nutritional status of adolescent in Ethiopia over the last 10 years.

**Fig 6:**
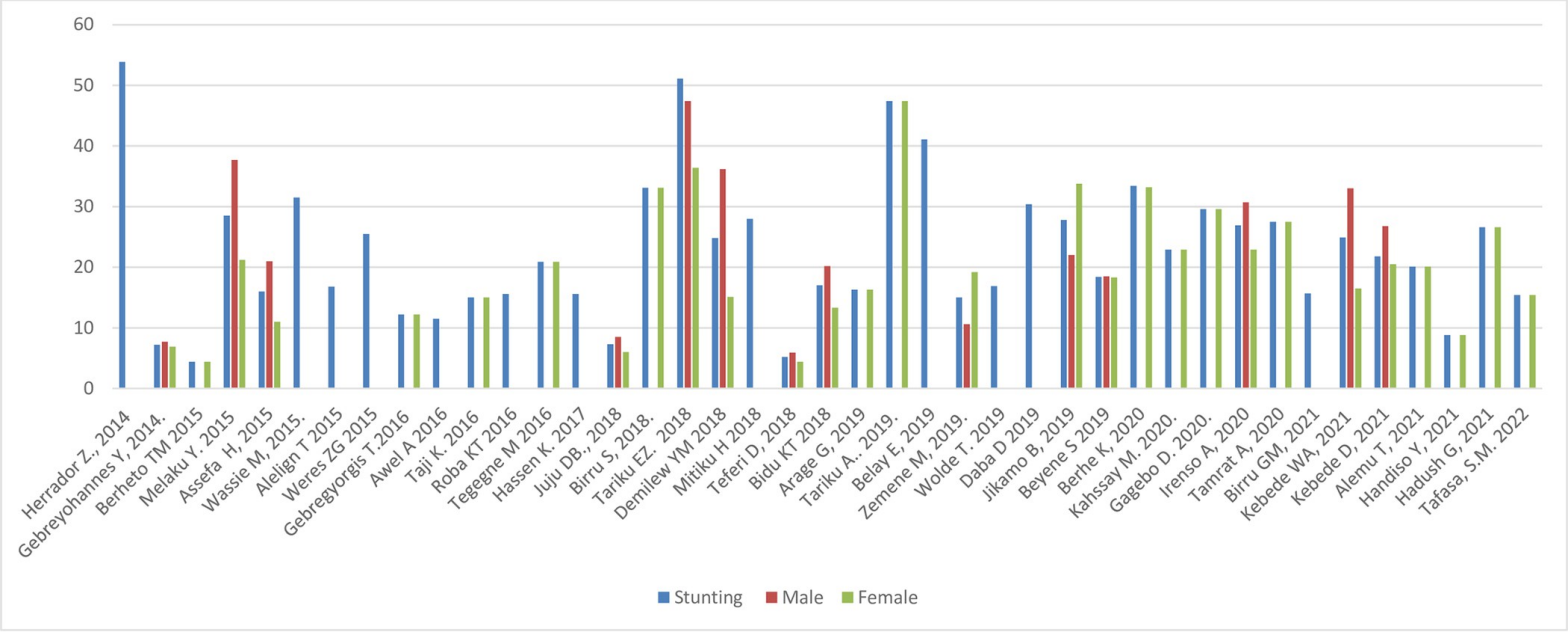
Trends in adolescent stunting by sex in Ethiopia over the last 7 years.

**Fig 7:**
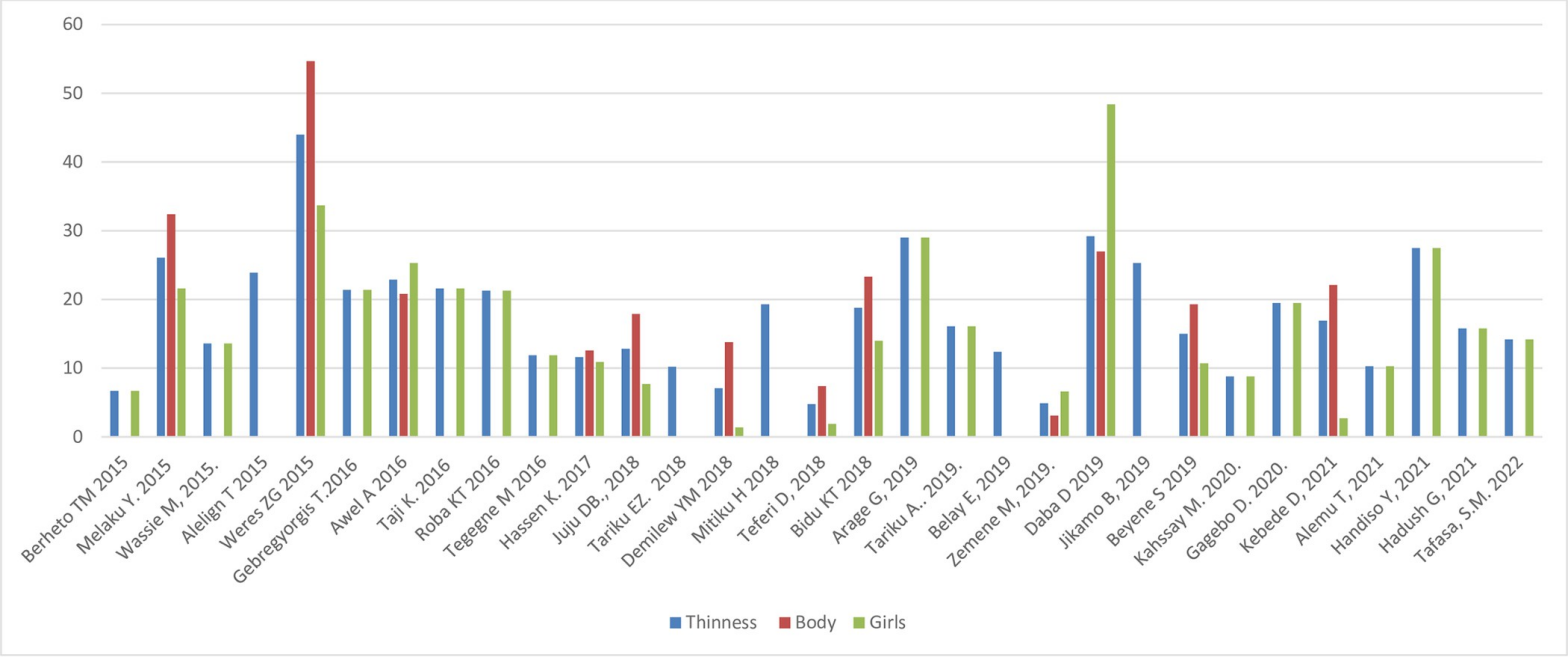
Trends in adolescent thinness by sex in Ethiopia over the last 6 years.

**Fig 8:**
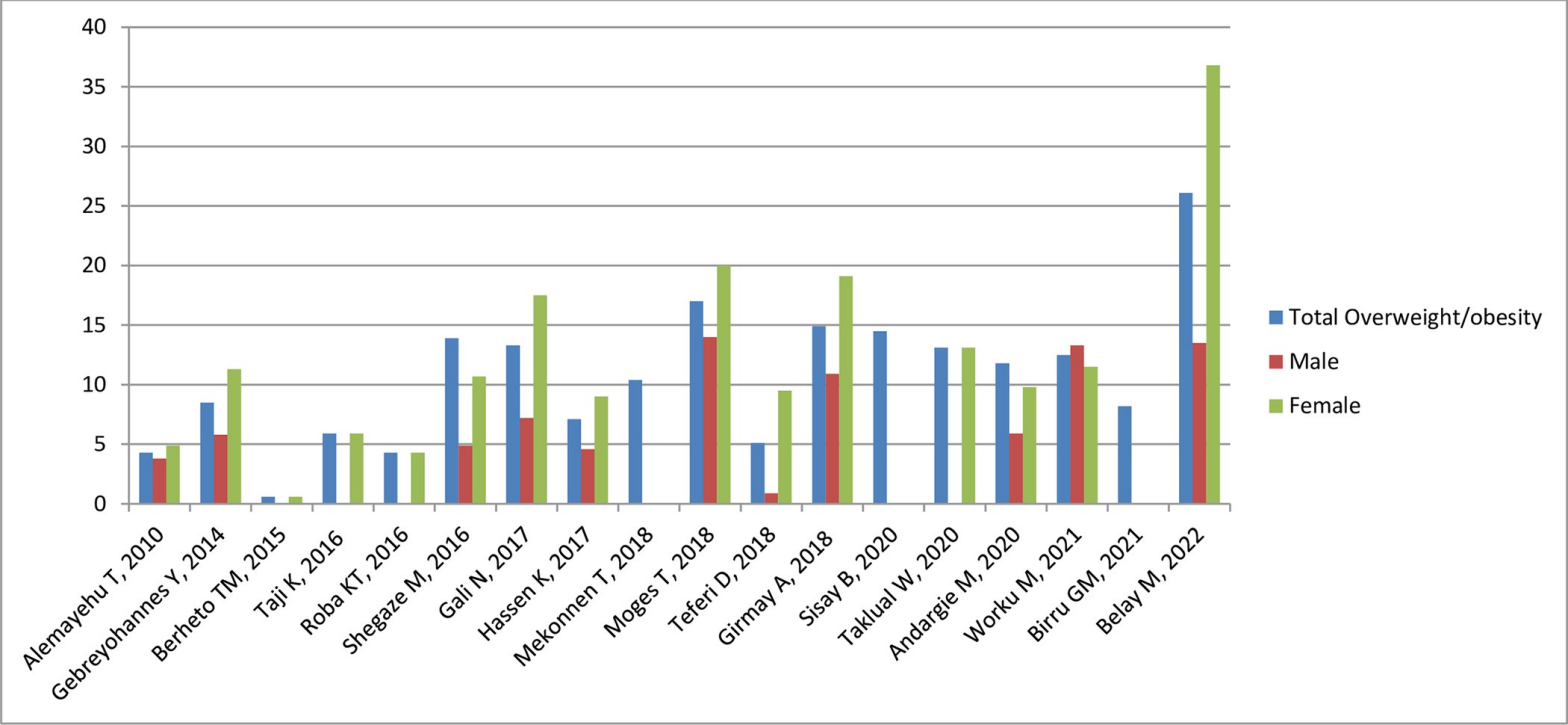
Trends in adolescent overnutrition by sex in Ethiopia over the last 12 years.

**Fig 9:**
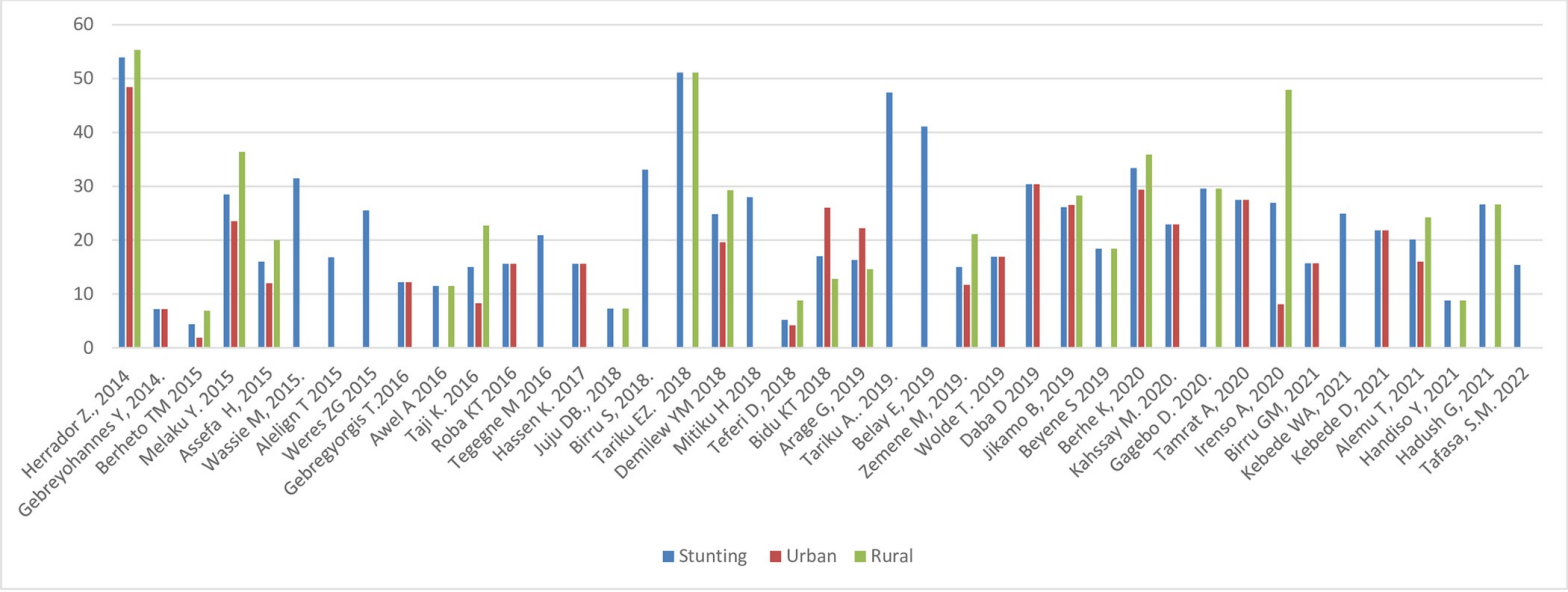
Trends in adolescent stunting by setting in Ethiopia over the last seven years.

**Fig 10:**
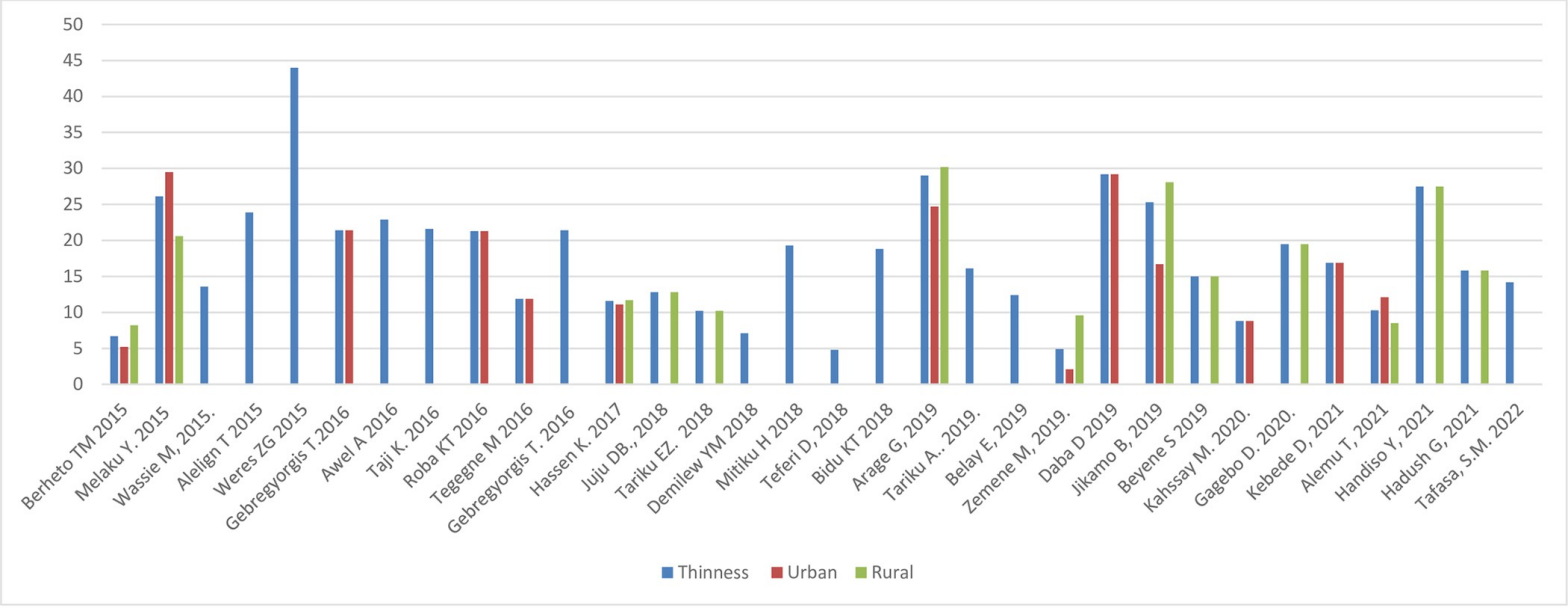
Trends in adolescent thinness by setting in Ethiopia over the last 6 years.

**Fig 11:**
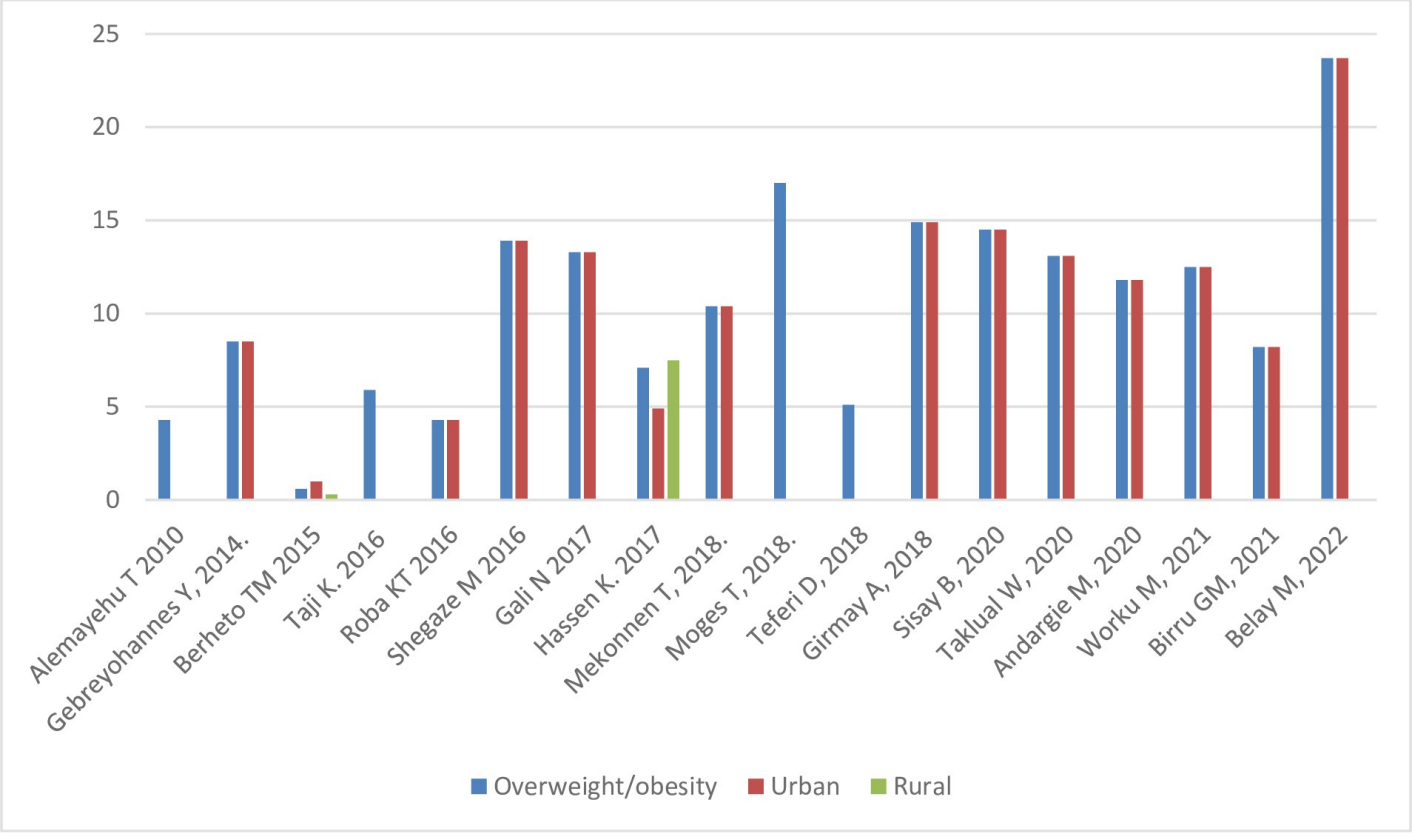
Trends in adolescent overnutrition by setting in Ethiopia over the last 10 years.

Fig 5 shows the prevalence of stunting, thinness, underweight and overweight/obesity across the studies included, arrayed by year of publication; these outcomes are also shown stratified by sex and setting (rural/urban) in Figs 6–11. Among studies that measured the prevalence of undernutrition, the prevalence of stunting ranged from 4.4% in girls (urban 1.9% & rural 6.9%) in southwest Ethiopia [32] and 5.2% (5.9% boys, 4.4% girls, 4.2% urban & 8.8% rural) in Wolaita Sodo (south Ethiopia) [41] to 53.9% (urban 48.4% & rural 55.3%) in a sample of both sexes from northwest Ethiopia [30]. In terms of sex, stunting ranged from 5.9% in boys and 4.4% in girls in Wolaita Sodo town [41] and 7.2% in boys and 6.9% in girls (total 7.7%) in Addis Ababa city [31] to 47.4% in boys and 47.4% in girls in rural northwestern Ethiopia [57]. The prevalence of thinness ranged from 4.9% (boys 3.1%, girls 6.6%) in central Northern Ethiopia [59] to 44% (54.7% in boys and 33.7% in girls) in eastern Tigray (north Ethiopia) [36]. Thinness, in terms of sex, ranged from 3.1% [59] to 54.5% [36] in boys, and from 1.4% [51] to 48.4% [61] in girls. In terms of setting, stunting ranged from 1.9% in urban and 6.9% in rural settings [32] to 48.4% urban and 55.3% rural settings [30]. The prevalence of overweight/obesity ranged from 0.6% in girls (urban 1% & rural 0.3%) in southwestern Ethiopia [32] to 17% (boys 14% & girls 20%) in Addis Ababa city [50].

In ten of the fourteen studies that reported stunting in both sexes, the prevalence was higher in boys [22, 31, 33, 36, 41, 46, 49, 51, 55, 63] while in four stunting was higher in girls [38, 45, 59, 62]. Nine of twelve studies, reported more thinness in boys than girls [33, 36, 41, 45, 46, 51, 55, 62, 63]. In all of the five studies that have reported overnutrition, the prevalence of overweight/obesity was higher in girls [29, 31, 41, 43–45, 50, 53], and in three of four studies that have reported under nutrition, the prevalence of underweight was higher in boys [29, 31, 36].

#### Nutritional status by age

In eight of fifteen and in nine of thirteen studies, the prevalence of stunting and thinness was higher in younger (10–14 years) compared to older (15–18 years) adolescents. The prevalence of stunting ranged from 2.1% [47] to 38.5% [64] in younger (10–13 years), and from 3.5% [46] to 61.7% [47] in older (16–18 years) adolescents. Thinness in younger adolescents ranged from 11% in Jimma zone, southwestern Ethiopia [45] to 64.3% early (10–13 years) in northwest Ethiopia [56], and in older adolescents it ranged from 3.8% in southwestern Ethiopia (Wollega Zone) [55] to 39.7% in eastern Ethiopia [52].

#### Nutritional status trends over time

We searched for individual articles published since 2000, but data on the nutritional status of adolescents was only available between 2010 and 2022 The data from individual studies showed no clear pattern across time (Figs 2–8); and both undernutrition and overnutrition have co-existed over the last 10 years. A high prevalence of stunting (54% and 51%) was documented in 2014 and 2018, respectively; while a prevalence of 80.8% underweight and 44% thinness was documented in 2015. Likewise, the highest prevalence of overweight (17%) was documented in 2018.

#### Predictors of nutritional status

In ten of thirteen studies, adolescents from rural areas were more likely to be stunted compared to urban adolescents [22, 30, 32, 33, 39, 41, 51, 59, 62, 64]. The prevalence of thinness was higher among adolescents from less educated mothers, adolescents who have <3 meals per day and those from households comprising more than five people. In addition, adolescents who were physically inactive and adolescents with sedentary lifestyles were more likely to be obese than others [44].

The reviewed articles [22, 30, 32, 33, 39, 41, 51, 59, 62, 64] also identified common predictors for under- as well as overnutrition. Low dietary diversity, low frequency of daily food intake, higher household family size, low maternal education, food insecurity, and poor quality sources of drinking water were associated with undernutrition. In contrast, residency in urban settings, female sex, low levels of physical activity and a more sedentary life style are predictors of overweight/obesity (Table 1).

**Table 1:** Studies reporting anthropometric measures.

First author, y	Main objective (s)	Study design	Setting: Rural/urban	Sample size	Age (y)	Sex (M/F)	Exposure (s)	Outcome (s)	Main findings
Alemayehu T, 2010 [29]	To assess the magnitude of adolescents’ undernutrition and its determinants in public schools	Cross-sectional study	Urban	425 in-school adolescents	10–19	M/F	Age, sex, food intake, family livestock ownership	Under-weight and over-weight	Underweight 27.5% (boys 29.8%, girls 24.6%), young adolescents 38.1%, older adolescents 18.6%) Overweight 4.3% (boys 3.8%, girls 4.9%) Underweight predictors: younger age between 10–14 years (AOR = 1.99, 955% CI: 1.01–3.57), household who produce inadequate food supply as a result obliged to purchase (AOR = 2.4, 95% CI: 1.24–4.74) and family possessed no cattle (AOR = 2.4, 95% CI: 1.24–4.74)P<0.05)
Gebreyohannes Y, 2014 [31]	To assess and compare nutritional status of adolescents and analyze the risk factors associated with overweight/obesity in government and private secondary schools	Comparative cross sectional study	Urban	1024	13–19	M/F	School type	Stunting, underweight, overweight/ obese	Stunting 7.2% (boys 7.7%, girls 6.9%, public school 10.0%, private 4.5%). Underweight 6.2% (boys 9.8%, girls 2.6%, public school 7.0%, private 5.5%), Overweight/obese 8.5% (boys 5.8%, girls 11.3%, public school 4.3%, private 12.7%). Adolescent in private schools are more overweight/obese (AOR 2.2; 95% CI: 1.2–4.2)
Herrador Z, 2014 [30]	To determine prevalence of stunting and thinness and their related factors in Libo Kemkem and Fogera, and compare urban and rural areas. Northwest Ethiopia	Cross-sectional Study	Urban/rural	886 children (259 aged 10–15)	11–15	M/F	Residence/setting	Stunting and thinness	Stunting 53.9% (rural 55.3% and urban 48.4%, P-value <0.05)
Assefa H, 2015 [22]	To identify socio-demographic factors associated with underweight and stunting among adolescents	5-year longitudinal study	Urban/rural	2084	Mean age 14.8 (SD 1.3)	M/F	Socio-demographic factors	Stunting and underweight	Stunted 16% (boys 21%, girls 11%, urban 12%, semi-urban 16%, rural 20%). Underweight 80.8% (boys 73%, girls 89%%, urban 83%, Semi urban 84%, rural 75%, p-value <0.05), Underweight predictors: male sex (β = -0.7; 95% CI: -0.8, -0.6), age in years (β = 0.1; 95% CI: 0.02, 0.1), attending public school (β = 0.8; 95% CI: 0.02, 1.6) Stunting predictors: male sex (β = -0.2; 95% CI: -0.3, -0.1), attending private school (β = —1.2; 95% CI: -1.9, -0.5), household income (β = 0.001, 95% CI: 0.001, 0.002), household size (β = -0.02, 95% CI: -0.04, -0.01)
Roba A, 2015 [90]	To assess nutritional status and dietary intake of rural adolescent girls and determine pulse and food intake patterns associated with poor nutritional status	Cross-sectional study	Rural	188	15–19	F	pulse and food intake patterns	Stunting and Underweight	Stunting was 30.9% and underweight was 13.3%. Stunting and underweight associated with low food and nutrient intake.
Melaku Y, 2015 [33]	To determine prevalence and factors associated with stunting and thinness	Cross sectional study	Rural/urban	348 School adolescents	10–19	M/F	Sex, sex & setting	Stunting and thinness	Stunting 28.5% (boys 37.7%; girls 21.2%, urban 23.5%, rural 36.4%) Thinness 26.1% (boys 32.4, girls 21.6%, urban 29.5%, rural 20.6%) Mean height-for-age and BMI-for-age Z-scores: -1.49 & -1.29, respectively. Stunting predictors: age 13–15 years (AOR = 2.23; 95% CI: 1.22, 4.08), being male (AOR = 2.53; 95% CI: 1.52, 4.21) and rural residence (AOR = 2.15; 95% CI: 1.20, 3.86). Thinness predictor: male sex (AOR = 1.97; 95% CI: 1.19, 3.25), age 16–19 years (AOR = 0.5; 95% CI: 0.2, 0.9) compared to age 10–12 years
Berheto TM, 2015 [32]	To determine urban-rural disparities in the nutritional status of school adolescent girls in the Mizan district, south-western Ethiopia	Comparative cross-sectional study	Urban/rural	622 (rural 311 and urban 311)	11–19	F	Setting	Stunting	Stunting 4.4% (urban 1.9% and rural 6.9%) Thinness 6.7% (urban 5.2% and rural 8.2%) Overweight 0.6% (urban 1% and rural 0.3%) Mean height-for-age Z-score and BMI-for-age Z-score: –0.6 ± (0.9) and –0.4 (1.0) in urban and –0.8 (0.8) and –0.5 (0.9) in rural areas, respectively
Weres ZG 2015 [36]	To assess the prevalence of adolescent under nutrition and its associated factors	Cross sectional study	Unstated	411	10–19	M/F	Age, sex,	Stunting, thinness, underweight	Stunting, 25.5% (Boys 29.6%, girls 21.6%), thinness 44% (boys 54.7%, girls 33.7%) and underweight 55% (boys 65.5%, girls 44.7%) Thinness predictors: younger (10–14 years) age AOR = 4.7; 95% CI = 1.8, 12.1), male sex (AOR = 5.3; 95% CI = 1.7, 16.3)
Wassie M, 2015 [34]	To assesses level of low BMI-for- age and height-for- age and their associated factors	Cross-sectional study	Unstated	1320	10–19	F	Age, dietary diversity, access for nutrition information, and community based nutrition service, food insecurity	Stunting, thinness	Stunting 31.5%, Thinness 13.6% Thinness predictors: age group 10–14 years (AOR = 5.8, 95% CI: 3.3, 10.4), age group 15–17 years (AOR = 2.1, 95% CI: 1.1, 3.9), with poor dietary diversity score (AOR = 2.5, 95% CI: 1.6, 3.8), utilizing community based nutrition service (AOR = 0.7, 95% CI: 0.5, 0.9) Stunting predictors: age group 10–14 years (AOR = 6.1, 95% CI: 4.0,9.2), age group 15–17(AOR = 1.4, 95% CI: 1.9,2.1), had nutrition and health information(AOR = 1.9, 95% CI: 1.5, 2.6), living in food secured households (AOR:0.7,95% CI: 0.5, 0.8)
Alelign T 2015 [35]	To assess the prevalence and factors associated with undernutrition	Cross sectional study	Urban/rural	403 (209 age 10–14)	10–14	M/F	-	Stunting	Stunting 16.8%, underweight 23.9%
Awel A, 2016 [38]	To assess nutritional status and associated factors	Cross sectional	Rural	655	10–18	M/F	Age, sex, family occupation, family size, parental education, daily food intake frequency	Stunting, thinness	Stunting 11.5% (boys 8.4%, girls 14.9%) Thinness 22.9% (boys 20.8%, girls 25.3%) Stunting predictors: female sex (AOR1 = 2.4, 95% CI: (1.3, 4.3); Age 15–18 (AOR = 10.9, 95% CI: 4.8, 24.4), Family size >5 (AOR = 1.9, 95% CI: 1.1, 3.6), lower family wealth index (AOR = 3.2, 95% CI: 1.5, 6.9), Food insecure adolescent (AOR = 2.6, 95% CI: 1.4, 4.9), agro pastoral family occupation (AOR = 2.5, 95% CI: 1.4, 4.7). Thinness predictors: family size >5 (AOR = 1.7, 95% CI: 1.1,2.6), lower family wealth index (AOR = 1.9 (AOR = 1.1, 95%CI:1.1, 3.2), food insecure adolescent (AOR = 2.0, 95%CI: 1.2,3.3)
DHS report: Adolescent nutrition, 2000–2016 [75]	To assess the nutritional status of adolescent	Cross-sectional survey	Urban/rural	-	15–19	M/F		Thinness	Thinness: girls = Urban 2.2%, rural 6.8%; Boys = Urban 22.9%, rural 29.6% Overweight: girls = Urban 11.4%, rural (not indicated)? BMI-for-age: Girls thin: 2000 (12.3%), 2005(9.4%), 2011 (8.7%), 2016 (5.7%) BMI-for-age: thinness boys: 2003 (36.6%), 2008 (28.3%) BMI-for-age: Girls overweight: 2000 (2.1%), 2005 (3.9%), 2011 (3.2%), 2016 (4.9%). BMI-for-age: boys overweight: 2003 (0.5)%, 2008 0.8%) Percentage of short stature girls: Urban (10.0%), rural (13.0%) BMI-for-age: Girls; 2000 (20.4%), 2005 (16.6%), 2011 (17.7%), 2016 (12.4%).
Roba KT, 2016 [40]	To identify the level of malnutrition and associated Factors	Cross sectional study	urban	726	15–19	F	Parental education, father occupation, DDS,	Stunting and thinness	Stunting 15.6%, Thinness 21.3%, Overweight 3.3%, obese 1.0%, Thinness predictors: Adolescent from illiterate mother (AOR = 5.4; 95% CI: 4.71–9.1, mothers primary level education (AOR = 1.7; 95% CI:0.9–3.2), FATHERS Illiterate (AOR = 3.1; 95% CI:1.7–5.6), Father primary level education (AOR = 2.4; 95% CI:1.4–4.0), Father ocuupation as daily laborer (AOR = 2.7;95% CI:1.5–4.8), adolescent low DDS (AOR = 2.1; 95% CI:1.5–3.9),
Tegegne M, 2016 [42]	To assess the nutritional status and associated factors	Cross sectional study	Urban/rural	598	10–19	F	Age, setting, parental education, parental occupation, family size, DDS	Stunting, thinness	Stunting 20.9%, thinness 11.9% Stunting predictors: mothers illiterate (AOR = 13; 95%CI: (2.7–18.08), low DDS (AOR = 2.7; 95% CI: 1.5–5.04) Thinness predictors: age≤14 (AOR = 1.7; 95%CI: 1.5–2.6), mother illiterate (AOR = 9.6; 95% CI: 2.6–23.3), mother only read/write (AOR = 7.6; 95% CI: 2.2–19.1), mother primary level education (AOR = 5.2; 95%CI:1.4–17.4)
Taji K, 2016 [39]	To assess the nutritional status of adolescent girls	Cross sectional study	Urban/rural	547	10–19	F	Setting, water source, parental education, parental occupation,	Stunting, thinness, overweight, obesity	Stunting 15% (95% CI: 12.1, 18.3) (urban 8.3%, rural 22.7%), Thin 21.6%, Overweight 4.8% (95% CI: 3.1, 6.)9 Obese 1.1% obese (95% CI: 0.4–2.3) Stunting predictors: fathers with farming occupation (AOR = 2.4; 95% CI: 1.2–4.8), rural residence (AOR = 0.4; 95% CI: 0.2–0.8), younger adolescent (AOR = 0.5; 95% CI: 0.3–0.9)
Shegaze M, 2016 [43]	To determine the prevalence of overweight/obesity and associated factors	Cross sectional study	Urban	456	13–19	M/F	Se, age, family wealth status, physical activity, nutrition knowledge	Overweight/obesity	Overweight 9.7% (95% CI: 6.9, 12.4%), Obesity 4.2% 95% CI: 2.3, 6.0%), Overweight/obesity 13.9% (95% CI: 10.6, 17.1%, boys 4.9%, girls 27.6%) Overweight/obesity predictors: female sex (AOR = 7.3; 95%CI: 3.8, 14.1), private school (AOR = 3.5; 95%CI: 2.0, 6.2), high family wealth (AOR = 4.8; 95%CI: 2.4, 9.8), day time sitting >3 hours (AOR = 6.1; 95%CI: 3.5, 10.8), family size>4 (AOR = 0.3; 95%CI: 0.2, 0.6), low total physical activity level (AOR = 8; 95%CI: 3.9, 16.2), ate sweet food in last 7 days (AOR = 6.3; 95%CI: 3.6, 10.9), meal >3times/day (AOR = 3.0; 1.4, 6.6), better nutrtion knowledge (AOR = 0.2; 95%CI: 0.1, 0.4),
Gebregyorgis T. 2016 [91]	To assess the prevalence of thinness, stunting, and associated factors	Cross sectional	Urban /rural	814	10–19	F	Age, mother education, eating frequency, poor water source, family size, father occupation, father education, wealth index,	Stunting and thinness	Stunting 12.2%, Thinness 21.4% Stunting predictor: Family size >5 [AOR = 2.05 (1.31, 3.23)] and unimproved source of drinking water [AOR = 3.82 (2.20, 6.62)] Thinness predictors: Age of adolescent [AOR = 2.15 (1.14, 4.03)], mother’s educational status [AOR = 2.34 (1.14, 4.80)], eating less than 3 meals per day [AOR = 1.66 (1.12, 2.46)], having family size >5 [AOR = 2.53 (1.66, 3.86)]
Gali N, 2017 [44]	To determine the prevalence and predictors of obesity and overweight among school adolescents in Jimma town	A school-based cross-sectional study	Urban	546	Mean age 15.37 (SD 1.88)	M/F	Age, sex, parental education, dietary intake, school type, family wealth, physical activity,	Overweight/Obesity	Overweight/obesity 13.3% (boys 7.2% and girls 17.5%). Overweight/Obesity predictors: female sex (AOR = 3.4; 95% CI:1.3–9.9]), attending private schools (AOR = 7.5; 95% CI: 2.5–22.3), adolescents from wealthy households (AOR = 3; 95% CI:1.1–8.3]) and. those who were physically inactive (AOR = 3.7; 95% CI:1.1–13.02]) and adolescent with sedentary lifestyles (AOR = 3.6; 95% CI:1.4–9.5) were found to be more obese than their counter peers.
Hassen K, 2017 [45]	To investigated the nutritional outcomes of adolescents and their determinants in coffee farming households	Cross-sectional study	Urban/rural	550	10–19	M/F	Age, residency, family wealth, age dependent family size, parental education, household food insecurity, family size,	Stunting, thinness, overweight/obesity	Stunting 15.6% (girls 16.0%, boys 15.1%, urban 19.8%, rural14.9%), Thinness 11.6% (girls 10.9%, boys 12.6%, urban 11.1%, rural 11.7%), Overweight/obesity 7.1% (girls 9.0%, boys 4.6%, urban 4.9%, rural 7.5%) Stunting predictors: lower teritial of wealth index (AOR = 5.6, 95% CI: 2.6–12.0), Overweight/obesity predictors: middle teritial of wealth index (AOR = 2.7; 95% CI 1.1–6.9) compared to highest wealth index teritial, adolescents in low age dependent family size of 1–2 person/household(AOR = 2.6; 95% CI:1.1–6.2), male sex (AOR = 2.4; 95% CI:1.1–5.1) Thinness predictors: lower wealth teritial (AOR = 5.9; 95% CI: 2.8–12.9), higher family size (AOR = 1.3; 95% CI:1.1–1.5)
Bidu KT 2018 [55]	To assess the prevalence and associated factors of undernutrition	Cross sectional study	urban/rural	640	10–19	M/F	-	Stunting, thinness,	Stunting 17.0% (95% CI; 14%, 20%, boys 20.2%, girls 13.7%, urban 26.0%, rural 12.8%) Thinness 18.8%(95% CI; 15.6%, 21.9%, boys 23.3%, girls 14%)
Birru SM, 2018 [47]	To assess prevalence of stunting and associated factors among school adolescent girls in Gondar City	Cross-sectional study	Urban	812	10–19	F	Age, type od school, parental education, parental occupation, dietary diversity, family wealth index and media exposure	Stunting	Stunting 33.1% (private school 12.1%, public school 38.8%) Stunting predictors: younger (AOR = 0.2; 95% CI: 0.0,0.2), middle age adolescent (AOR = 0.2; 95% CI: 0.2, 0.3), and unsatisfactory media exposure (AOR = 1.7; 95% CI: 1.1, 2.8) and poor mother’s education (AOR = 2.8; 95% CI: 1.1, 7.9)
Juju D, 2018 [46]	To assess prevalence and factors associated with nutritional status of adolescents in the selected khat and coffee-growing areas	Cross-sectional study	Rural	234	12–18	M/F	Health problems in the past 30 days	Food insecurity experiences	Stunting 7.3% (boys 8.5%, girls 6.0%). Thinness 12.8% (boys 17.9%, girls 7.7%). Stunting predictors: age 12–14 years (AOR = 3.6; 95% CI, 1.1, 11.5), adolescent from illiterate mothers (AOR = 5.6; 95% CI, 1.6, 20.4). Thinness predictors: Female sex (AOR = 0.4; 95% CI, 0.2, 0.9), dietary frequency <3 times a day (A OR = 4.164; 95% CI, 1.6, 10.7)
Teferi D, 2018 [41]	To assess the prevalence of malnutrition and associated factors	Cross sectional study	Urban/rural dominated by urban	655	10–19	M/F	Age, sex, maternal education, DDS, school type	Stunting, thinness, overweight	Mean height 162.43 cm and weight51.96 kg. Mean HAZ −0.49, and BAZ −0.58 Stunting 5.2% (95% CI: 3.4%,7%, boys 5.9%, girls 4.4%, urban 4.2%, rural 8.8%), thinness 4.8% (95% CI: 3%,6.7%, boys 7.4%, girls 1.9%), and overweight/obesity 5.1% (boys 0.9%, girls 9.5%) Stunting predictors: Maternal secondary educational level (AOR = 0.2; 95% CI: 0.1, 0.9) Thinness predictors: Being male (AOR = 4.1; 95% CI: 2.4,7.0), adolescent from public school (AOR = 0.4; 95% CI: 0.2,0.7), mothers with no formal education (AOR = 4.0; 95% CI: 1.8,8.9), skipping meals (AOR = 1.7; 95% CI: 1.1, 2.7), and illness in 2 weeks prior to survey (AOR = 2.7; 95% CI: 1.5, 4.8) Overweight/obesity predictor: being male (AOR = 0.1; 95% CI: 0.03, 0.2)
Zenebe M, 2018 [92]	To examine the effects of school feeding program on dietary diversity, nutritional status and class attendance of school children	Comparative cross-sectional study	Urban/rural	292	10–14	M/F	School food program	HAZ, BAZ, DDS	Mean (±SD) HAZ score in adolescents with school feeding program was (− 1.45 ± 1.38) compared to those without school feeding program (− 2.17 ± 1.15 which was statistically significant (P < 0.001) adjusted for age, sex, family wealth and parental educational status.
Tariku E, 2018 [49]	To assess the prevalence of stunting and thinness and their associated factors among school age children	cross-sectional study	Rural	389 (137 aged 12–14)	12–14	M/F	-	stunting and thinness	Stunting 51.1% (boys 47.4, girls 36.4%), Thinness 10.2%.
Mekonnen T, 2018 [48]	To assess the prevalence of overweight/obesity and associated factors	cross-sectional study	Urban	634 (327 aged 10–14)	10–12	M/F	-	Overweight/obesity	Overweight/obese 10.4%
Moges T, 2018 [50]	To determine and compare the levels of overweight/obesity among adolescents in private schools with and without adequate play area	Cross-sectional study	Urban	1,276	10–19	M/F	School play area	Obesity	Overweight/obesity 17.0% (boys 14%, girls 20%, age 10–14 years 16.8%, age 15-19years 17.3%). Mean ± SD BAZ was −0.2± 1.3 Overweight predictor: School with no adequate play area (AOR = 1.6; 95% CI: 1.1, 2.5)
Mitiku H, 2018 [52]	To assess the nutritional status of adolescent	Cross sectional study	urban/rural	1523 (767 aged 10–18)	10–18	M/F	-	Stunting and thinness	Stunting 28.0% (in age 10–14 = 26.0%, age 15–18 = 35.3%) Thinness 19.3%(in age 10–14 = 17.6%%, age 15–18 = 39.7%)
Girmay A, 2018 [53]	To assess the prevalence of overweight, obesity and associated factors	Cross sectional study	Urban	950	12–15	M/F	Age, sex, family size, family income, dietary intake,	Overweight/obesity	Overweight/obesity 14.9% (boys 10.9%, 19.1%) positive predictors are female sex (AOR = 1.8; 95% CI:1.2, 2.6)) and taking soft drinks four or more times per week(AOR = 1.0;95%CI: 0.4, 4.6) and lower (<4) family size (AOR = 3.0;95%CI;1.9, 5.0)
Demilew Y, 2018 [51]	To assess the prevalence of under nutrition and its associated factors	cross-sectional study	Urban /rural	424 school adolescents	Mean 16.7 (SD 0.9)	M/F	Sex, parental residence, frequency of dietary intake, water source, family size, illness episode	Under nutrition (stunting and thinness)	Stunting 24.8% (boys 36.2%, girls 15.1%, urban 19.6%, rural 29.3%) Thinness 7.1% (boys 13.8%, girls 1.4%) Stunting predictors: Male sex (AOR = 3.2; 95% CI: (1.7, 5.8), low dietary frequency (1–2 times per day) (AOR = 4.6; 95% CI: 2.6, 8.0), lack of latrine (AOR = 2.7, 95% CI: 1.2, 6.0), and poor hand washing practice (AOR = 3.9; 95% CI: 1.9, 8.1). Thinness predictors: being male [AOR = 11.5; 95% CI: 3.3, 39.5), illness in the last two weeks (AOR = 2.9; 95% CI: 1.2, 7.0), and having more than five family members (AOR = 3.6; 95% CI: 1.3, 9.4)
Tariku EZ 2018	to assess the prevalence of stunting and thinness and their associated factors	Cross-sectional	Rural	389 (age 12–14, n = 137)	12–14	M/F	Sex, age, family size, family income, food security, DDS, parental education,	Stunting and thinness	Stunting 51.1% (boys 47.4%, girls 36.4%) Thinness 10.2%
Arage G, 2019 [56]	To determine the prevalence and factor associated with nutritional status of school adolescent girls in Lay Guyint Woreda, Northwest Ethiopia	Cross-sectional study	Urban /rural	362	10–19	F	Age, residence, mother’s occupation, dietary diversity, frequency of dietary intake	Stunting and thinness	Stunting 16.3% (urban 22.2%, rural 14.6%). Thinness 29% (urban 24.7%, rural 30.2%). Stunting predictors: aged 14–15years (AOR = 3.7; 95% CI: 1.9, 7.1), residence in rural areas (AOR = 1.3; 95% CI: 1.2, 2.3), those who did not have snack (AOR = 11.4; 95% CI: 1.5, 17.8) and farming mother’s occupation (AOR = 0.1; 95% CI: 0.2, 0.9). Thinness predictors: rural resident (AOR = 2.4; 95% CI: 1.1, 5.1) and adolescents aged 14–15years (AOR = 6.1; 95% CI: 2.2, 17.1).
Belay E, 2019 [58]	To find out the prevalence and determinants of pre-adolescent (5–14 years) acute and chronic undernutrition	Cross sectional study	Urban/rural	848 (338 aged 10–14)	10–14	M/F	-	Stunting and thinness	Stunting 41.1%, thinness 12.4%
Beyene S 2019 [63]	To assess the prevalence of undernutrition and associated factors	Cross sectional study	Rural	1437	10–19	M/F	-	Stunting	Stunting 18.4% (boys 18.5%, girls 18.3%) and thinness 15.0% (boys 19.3%, girls 10.7%)
Daba D, 2019 [61]	To assess the prevalence of undernutrition and its associated factors	Cross sectional study	Urban	312	12–18	M/F	Age, sex, DDS, food intake frequency, water source, substance use,	Stunting and thinness	Stunting 30.4%, thinness 29.2% (Boys 27.0%, girls 48.4%), Thinness predictor: female sex (AOR: 2.55; 95%CI: 1.16–5.63), Ever skipped one or more daily meal per day (AOR: 6.56; 95%CI: 2.25–19.15), low dietary diversity score (AOR: 1.86; 95%CI: 1.05–3.27) and using unprotected water source (AOR: 1.78;95%CI: 1.03–3.05) Stunting predictors; age group 15–18 (AOR: 5.78; 95%CI: 3.20−10.40) and ever used substance (AOR: 3.01; 95%CI: 1.17–7.77).
Jikamo B, 2019 [62]	To assess the association between dietary diversity and nutritional status of adolescents	Cross sectional study (Data from the Jimma Longitudinal Family Survey of Youth (JLFSY)	Urban/rural	2084	13–17	M/F	Age, sex, household food insecurity, adolescent food insecurity DDS, workload	Stunting, thinness	Stunting 27.8% (boys 22%, girls 33.8%, Urban 26.5, Rural 28.3), Thinness 25.3% (urban 16.7%, 28.1%)) Stunning predictors: female sex (AOR = 2.0; 95% CI: 1.6, 2.4), household food insecurity (AOR = 1.7; 95% CI: 0.6, 0.9) Thinness predictor: Household food insecurity (AOR = 1.8; 95% CI: 0.6, 0.8), Rural residents (AOR = 1.6; 95% CI: 1.3, 2.2), Adolescent with higher workload (AOR = 2.6; 95% CI: 1.2, 3.1)
Tariku A, 2019. [57]	To assess the prevalence and associated factors of dietary diversity among adolescent girls.	cross-sectional study	Urban/rural	1550	10–19	F	-		Stunting 47.4% and thin 16.1%
Wolde T, 2019 [60]	To determine the prevalence of stunting and its impact on academic performance	Cross sectional study	Rural/urban	408 school adolescent	10–15	M/F	-	Stunting	Stunting 16.9%.
Zemene M, 2019 [59]	To assess the prevalence and its associated factors of nutritional status	Cross-sectional study	Urban/rural	327	10–19	M/F	Age, sex, residence, family size, water source		Stunting 15% (boys 10.6%, girls 19.2%, urban 11.7%, rural 21.1%). Thinness 4.9% (boys 3.1%, girls 6.6%, urban 2.1%, rural 9.6%). Stunting predictors: female sex (AOR = 2.2, 95% CI: 1.2, 4.4), rural residence (AOR = 2.5, 95%CI: 1.3, 4.8), and family size of ≥6 (AOR = 3.4, 95% CI:1.7, 7.1) Thinness predictors: Female sex (AOR = 1.8 95% CI: 0.5, 6.5), Rural residence (AOR = 3.7, 95% CI: 1.2, 11.6)
Berhe K, 2020 [64]	To assess the prevalence of undernutrition and associated factors among adolescent girls in Hawzen woreda, Northern Ethiopia	Cross sectional study	Urban/rural	398	10–19	F	Age, residence, parental occupation, parental education, frequency of dietary intake, family wealth	Stunting, underweight	Stunting 33.4% (urban 29.4%, rural 35.9%), Underweight 32.2% (urban 25.5%, rural 36.3%), Both stunted and underweight 8.8%. Underweight predictors: rural residence (AOR = 1.2; 95% CI: 0.3, 3.1), age 10–13 years (AOR = 0.6; 95% CI: 0.2, 1), unemployed father (AOR = 8.1; 95% CI: 0.5–12.5), unemployed mother (AOR = 2.4; 05% CI: 1.2, 3.6), father illiterate (AOR = 1.4; 95% CI: 1.1, 1.7) Stunting predictors: unemployed father (AOR = 3.2; 95% CI: 1.93–6.4), unemployed mother (AOR = 2.2, 95% CI: 1.1, 3.3), father illiterate (AOR = 1.6; 95% CI: 1.01, 2.2)
Gagebo D, 2020 [66]	To assess the prevalence of undernutrition and associated factors among adolescent girls	Cross-sectional study	Rural	719	10–19	F	Age, family size, parental occupation, parental education, family wealth and dietary frequency	Stunting and thinness	Stunting 29.6% (younger adolescent 25.7%, older 35.6%). Thinness 19.5% (younger adolescent 17.9%, older 21.8%). Stunting predictors: older adolescents (AOR = 2.1; 95% CI: 1.1, 3.9), farmer mother (AOR = 2.4; 95% CI: 1.3, 4.3) and employed mother (AOR = 3.1; 95% CI: 1.4, 6.9)), low household wealth index (AOR = 1.9; 95% CI: 1.3, 2.9), secondary maternal education ((AOR = 0.5; 95% CI: 0.3,0.9), and above secondary maternal education (AOR = 0.3; 95% CI: 0.1, 0.7)). Thinness predictors: father primary education ((AOR = 0.5; 95% CI: 0.3, 0.8) and fathers secondary education (AOR = 0.5; 95% CI: 0.3, 0.8), mother primary education (AOR = 0.6; 95% CI: 0.4, 0.9), adolescent having meal frequency (<2/day) (AOR = 1.9; 95% CI: 1.1, 3.1).
Kahssay M, 2020 [65]	To assess the nutritional status of adolescent girls and its associated factors	Cross-sectional study	Urban	348	10–19	F	Age, family size, dietary diversity, parental occupation,		Stunted 22.9%, thinness 8.8%. Stunting predictors: adolescent age 14–15 years (AOR = 1.4, 95% CI: 1.1–4.3), and dietary diversity score of <4 food groups (AOR = 2.2, 95% CI: 1.4–4.5). Thinness predictors: dietary diversity score of <4 food groups (AOR = 1.8, 95% CI: 1.1–4.4) and low food consumption (AOR = 3, 95% CI:1.2–7.9)
Taklual W, 2020 [93]	Aimed at assessing nutritional status and associated factors among female adolescents	school-based cross-sectional study	Urban	682	14–19	F	Age, family size, religion, ethnicity, parental occupation, parental education, family wealth, types of staple diet, diet diversity, menarche onset	Underweight, overweight, and obesity	Underweight 15%, overweight 8.4%, and obesity 4.7% Underweight predictors: Age groups of 14–16.5 years (AOR: 1.7, 95% CI: 1.03–2.69), family size ≥ 4 (AOR: 2.8, 95% CI: 1.05–4.99), participants who did not eat meat once per week (AOR: 1.6, 95% CI: 1.90–2.82), and no onset of menarche (AOR: 4.4, 95% CI: 1.21–15.75) Overweight predictors: family monthly income above 6500 ETB (AOR: 12.7, 95% CI: 2.47–65.62), consumption of meat two or more times per week (AOR: 2.07, 95% CI: 1.47–9.14), and consumption of fruit at least once a week (AOR: 0.20, 95% CI: 0.05–0.78)
Irenso A, 2020 [94]	To assess the magnitude and factors associated with adolescent linear growth and stunting	Cross-sectional	Urban/rural	2010	10–19	M/F	Age, sex, residence, hygiene,	Linear growth and stunting	Overall stunting 26.9% (Boys 30.7, girls 22.9; Urban 8.1%, Rural 47.9%. Significant interaction between residence and sex on the risk of stunting [AOR = 4.17 (95% CI 2.66, 9.9), P < 0.001], and height-for-age z score (HAZ) (b = 0.51, P < 0.001). In urban adolescents, older age (18 to 19 years) was negatively associated with linear growth (b = 0.29; P < 0.001). In rural setting, hand washing practice after toileting was positively associated with HAZ (0.62; P < 0.001) and with lower risk of stunting [AOR = 0.51 (95% CI 0.34, 0.76)]. Urban females had significantly higher HAZ than urban males [b = 0.52; P < 0.01)], and a significantly lower risk of stunting [AOR = 0.29 (95% CI 0.18, 0.48)].
Tamrat A, 2020 [95]	Aimed at determining the prevalence of stunting and its associated factors	school-based cross-sectional study	Urban	662	10–14	F	Age, religion, grade level, parental education, parental occupation, family size	Stunting	Stunting 27.5%. Stunting predictors: being grade 5 student [AOR; 95% CI: 1.90; 1.13–3.20], less than three meal a day [AOR; 95% CI: 2.37; 1.60–3.50], household food-insecurity [AOR; 95% CI: 2.52; 1.70–3.73]. Stunting preventive factors: Government employed mothers [AOR; 95% CI: 0.48; 0.26–0.89] or merchants [AOR; 95% CI: 0.43; 0.28–0.67]
Andargie M, 2020 [96]	to assess the magnitude and associated factors of overweight and obesity among public and private secondary school adolescents in Mekelle city	school-based comparative cross-sectional between private and public school adolescents	Urban	858	14–19	M/F	Age, type of school, religion, family size, birth order, grade level, physical activity, food frequency, type of transport to school, nutrition knowledge, parental occupation, parental education, parental wealth	Overweight and obesity	Overall overweight and obesity 7.8% (boys 5.9(, girls 9.8%, private school 11.8% and public schools 3.9%) Overweight/obesity predictors: Consuming dinner not daily [AOR = 5.3:95% CI = 1.93–14.6] and working moderate-intensity sports at least 10 minutes/day continuously [AOR = 0.19:95% CI = 0.04–0.9] were associated factors of overweight and obesity in public school adolescent students. Being female [AOR = 2.03:95% CI = 1.08–3.8], time taken from home to public physical activities ≤ 15 minutes [AOR = 3.6:95% CI = 1.13–11.51], using transport from school to home [AOR = 2.2:95% CI = 1.06–4.18] and good knowledgeable adolescents [AOR = 0.5:95% CI = 0.27–0.9] were associated factors of overweight and obesity in private schools.
Sisay B, 2020 [89]	To evaluate the performance of MUAC to identify overweight (including obesity) in the late adolescence period	Cross-sectional study	Urban	851	15–19	M/F	-		Overweight 11.2% (95% CI; 9.2–13.5%), Obesity 3.3% (95% CI; 2.3–4.7%) BMI Z score 0.44 (±1.2)
Worku M, 2021 [97]	To assess the prevalence and associated factors of overweight and obesity	nstitution-based cross-sectional study	Urban	551	10–19	M/F	Age, sex, school type, DDS, religion, parents occupation, family wealth status	overweight and obesity	Mixed overweight and obesity 12.5% (Boys 13.3%, Girls 11.5%) Overweight/obesity predictors: Having self-employed mothers (AOR: 4.57; 95% CI: 1.06, 19.78), having government-employed mothers (AOR: 6.49; 95% CI: 1.96, 21.54), and having school feeding access (AOR: 0.44; 95% CI: 0.26, 0.76)
Kebede D, 2021 [98]	To assess the prevalence and associated factors of stunting and thinness	school-based cross-sectional study	Urban	397	10–19	M/F	Age, sex, family wealth, grade, place of residence, religion, parental education, parental occupation, family size, DDS	Stunting and thinness among	Stunting 21.8% (Boys 26.8%, girls 20.5%) & thinness 16.9% (Boys 22.1%, girls 2.7%) Stunting predictors: having a family monthly income of less than $28.37 (P = 0.044) and having less than four dietary diversity (P = 0.021) Thinness predictors: Early adolescent age, being male, having a family monthly income of less than $28.37, having a family monthly income between $28.37 and $56.74 (P = 0.021) (35.25 Birr = 1 USD) and using clean water (P = 0.045)
Alemu T, 2021 [99]	Aimed at comparing the rural and urban prevalence’s ofstunting and thinness and their associated factors	ommunity-based comparative cross-sectional study	Urban/Rural	792	10–19	F	Age, educational status, residence, parental occupation, parental education, famlily sixe, family wealth, religion	Stunting and thinness	Stunting 20.1% (Urban 16%, Rural 24.2%), Thinness 10.3% (Urban 12.1%, rural 8.5%) Stunting predictors: Food insecurity [AOR: 1.95 (95% CI: 1.01, 3.78)] Stunting predictors in urban settings: early age adolescent [AOR:3.17 (95% CI:1.445,6.95)] Stunting predicators rural settings: lack of latrine [AOR: 1.95 (95% CI: 1.11, 3.43)], lowest media exposure [AOR: 5.14 (95% CI: 1.16, 22.74)], lower wealth class [AOR:2.58 (95% CI: 1.310, 5.091)], and middle wealth class[AOR: 2.37 (95% CI: 1.230, 4.554)] Thinness predictors Rural settings: Middle age adolescent groups [AOR: 3.67 (95% CI: 1.21, 11.149)]. Thinness predictors Urban setting: early age adolescent [AOR: 8.39 (95% CI: 2.48–28.30)].
Handiso Y, 2021 [100]	To assess the nutritional status and associated factors among adolescent	community-based cross-sectional study	Rural	843	10–19	F	Age, religion, school grade, family size, family income nutrition edutaion, dewarming, nutrtion service receved,	Thinness, stunting	Stunting 8.8%, Thinness 27.5%, Predictors of thinness: [AOR; 95% CI = 2.91; 2.03–4.173], large family size [AOR; 95% CI = 1.6; 1.11–2. 40], low monthly income [AOR; 95% CI = 2.54; 1.66–3. 87], not taking deworming tablets [AOR; 95% CI = 1.56;1.11–21], low educational status of the father [AOR; 95% CI 2.45; 1.02–5.86], source of food only from market [AOR; 95% CI = 5.14; 2.1–12.8], Predictors of stunting: lack of service from health extension workers [AOR; 95% CI = 1.72; 1.7–2.4], and not washing hand with soap before eating and after using the toilet [AOR; 95% CI = 2.25, 1.079–4.675]
Hadush G, 2021 [101]	to assess prevalence of nutritional status and associated factors among adolescent girls	school-based cross-sectional study	Rural	736	10–19	F	Age, family size, parents occupational status, parents educational status, family wealth status, household food insecurity	Thinness and stunting	Stunting 26.6%, Thinness 15.8%, Stunting predictors: being at an early adolescent age (AOR = 1.96, 95% CI 1.02–3.74), household food insecure (AOR = 2.88, 95% CI 1.15–7.21), menstruation status (AOR = 2.42, 95% CI 1.03–5.71), and availability of home latrine (AOR = 3.26, 95% CI 1.15–4.42). Thinness predictors: early age adolescent (AOR = 2.89, 95% CI 1.23–6.81)
Kebede WA, 2021 [102]	aimed at assessing the magnitude of stunting and associated factors among adolescent students	School survey	Urban/rural	424	14–19	M/F	Age, sex, religion, residence, family economy, parental education, grade level, water and sanitation,	Stunting	Stunting 24.9% (Boys 33%, girls 16.5%) Stunting predictors: male sex [AOR = 2.1; 95% CI: 1.73–5.90], meal frequency (<3/day) [AOR = 4.6; 95% CI: 2.61–8.24], infrequent hand washing practice [AOR = 3.6; 95% CI: 1.30–9.40], absence of latrine facility (AOR = 5.51; 95% CI: 3.03–9.9), and consumption of unsafe water [AOR = 2.8; 95% CI: 1.35–6.19].
Birru GM, 2021 [103]	to assess malnutrition and the associated factors among adolescents	School survey	Urban	365	14–19	M/F	Age, sex, parental marital status, DDS, food frequency, diet quality, mother occupation, snack intake	Stunting, underweight,/thinnessoverweight/obesity	Stunted 15.7%, Underweight 6.3%, and overweight/obesity 8.2%. Stunting predictor: Daily snack intake (AOR = 0.38, 95% CI: 0.20, 0.71), and inadequate diet quality (AOR = 3.36, 95% CI: 1.15, 7.82) underweight/thin: Being a male (AOR = 2.76, 95% CI: 1.03, 7.44) and meal consumption <3 times/day (AOR = 4.21, 95% CI: 1.35, 13.11) Overweight/Obesity: Dietary diversity score<5 (AOR = 0.35, 95% CI: 0.13, 0.89)
Kedir S, 2022 [104]	Aimed at identifying context-specific determinants of overweight and/or obesity among adolescents	School-based unmatched case-control study design	Urban	297	10–19	M/F	Sex, Age, wealth, soft drinks consumption, physically activity, screen time, nutritional knowledge, family size, parental education, diet diversity, fast food consumption	Overweight/ obesity.	High socioeconomic status [AOR = 5.8, 95% CI (2.66, 12.5)], consumed soft drinks 3and more times per week [AOR = 3.7, 95% CI (1.8, 7.3)], physically inactive [AOR = 4.4 95% CI (1.68, 11.6)], spent free time by watching television/ movies for 3and above hours per day [AOR = 8.6, 95% CI (4.3, 17)] and with poor nutritional knowledge [AOR = 3.4, 95%CI (1.7, 6.9)] were significantly associated with overweight/ obesity.
Tafasa, S.M. 2022 [105]	to assess the prevalence of undernutrition and its associated factors among school adolescent girls	School based study	Urban/rural	587	10–19	F	Age, religion, place f residence, family marital status, educational level parental education, family size, physical activity, DDS, food frequency, water source, nutrition knowledge, illness episodes, menstruation	Stunting and thinness	Stunting 15.4%; thinness 14.2%, Stunting predictors: Less than 3 meal/day [AOR = 3.62, 95% C.I (2.16, 6.05)], attending lower grades [AOR = 2.08, 95% C.I (1.07, 4.04)] and did not started menstruation [AOR = 1.71, 95% C.I (1.06, 2.73)] Thinness predictors: vigorous physical activities [AOR = 2.51, 95% C.I (1.14, 5.54)], low dietary diversity score [AOR = 4.05, 95% C.I (1.43, 11.46)] and younger adolescent (10–14 yrs) [AOR = 3.77, 95% C.I (1.06, 13.37)]
Belay M, 2022 [106]	To determine the magnitude of overnutrition and associated factors among school adolescents in Diredawa city	School based survey	Urban	498	10–19	M/F	Age, sex, meal preference, type of school, snack intake, physical activity, parental education, parental occupation, household wealth status	Overnutrition	Overnutrition of 26.1% (boys 13.5%, girls 36.8%); Overweight 23.7% and Obesity 2.4% Overnutrition Predictors: Being female (AOR = 3.32; 95% CI: 1.65–6.63), attending at private school (AOR = 4.97; 95% CI: 1.72–14.35), having sweet food preferences (AOR = 6.26; 95% CI: 3.14–12.5), snacking (AOR = 3.05; 95% CI: 1.11–8.36), sedentary behavior (AOR = 3.20; 95% CI: 1.67–6.09), and eating while watching TV (AOR = 2.95; 95% CI: 1.47–5.95)

#### Micronutrient deficiencies

Thirteen studies [39, 67, 68, 70–74, 76–80], on a sample of 7 019 adolescents, and one national micronutrient survey [69] on a sample of 722, and the DHS [75] on adolescent nutrition reported micronutrient status. The national micronutrient survey reported a mean Vitamin A concentration (retinol) of 1.20 μmol/l and 6.3% with retinol <0.7 μmol/l in the age group 12–14 years; equivalent data for the 15–19 year age group were 1.40±0.43, and 3.2% [69]. In the same survey for the same age group, zinc deficiency was found in 38%, Vitamin B12 deficiency (<203 pg/ml) in 13.6%, severe iodine deficiency (<20 μg/L) in 1.9%, moderate iodine deficiency (50–99.9 μg/L) in 25.2% and mild iodine deficiency (20–49.9 μg/L) in 20.5%. Excess iodine (>300 μg/L) was 12.2% in 12–14 years age groups [69].

Based on the micronutrient survey, the national prevalence of anaemia was 14.9% (14.4% moderate (Hb 8–12 g/dL, 0.5% severe (Hb<8g/dL) in adolescents aged 12–14 years and 11.8% (10.5% moderate and 1.3% severe) in the age group 15–19 years [69]. Iron deficiency (ferritin<15 μg/L) was 8.6% and iron deficiency anemia (IDA) was 2.6% in the age group 12–14; equivalent data for the 15–19 year age group were 10.0% and 3.2% [69].

Site specific studies in northwestern Ethiopia indicated a prevalence of anaemia of 13.4% (Hb *<* 12 g/dl [68] and 25.5% (92.4% mild, 5.9% moderate and 1.7% severe) with the odds of having anaemia higher in those with inadequate diet diversity score (DDS) (AOR = 2.1;95% CI; 1.3, 3.5) [71]. However, a study from eastern Ethiopia reported a prevalence of anaemia of 32% (HGB<12), 1.8% severe (Hb<7), 3.8% moderate (Hb 7–9.9), and 26.3% mild (Hb 10–11.9) [39].

The national prevalence of anaemia by sex and setting was similar (20.4%) for rural girls and boys while it was 16.7% for girls and 8.6% for boys in urban settings. The national trends in anemia prevalence in girls aged 15–19 years between 2000 and 2016 showed a steady reduction: 23.6% (1.0% severe, 12.7% moderate, and 9.8% mild) in 2005, 13.3% (1.0% severe, 4.7% moderate, and 7.5% mild) in 2011, and 19.6% (0.9% severe, 7.3% moderate and 11.4% mild) in 2016. Equivalent data for boys were 17.7% (0.4% severe, 2.4% moderate and 14.9% mild) in 2011 and 18.2% (severe 0.1%, moderate 3.6%, and mild 14.4%) in 2016 [75].

Two studies in Adama city (Central Ethiopia) by the same authors reported that the average serum vitamin D (25(OH)D) was 54.5nmol/L [73] and the prevalence of vitamin D deficiency (serum 25(OH)D <50 nmol/L) was 42% (female 51.5%, male 29.3%) [70]. Females (AOR1.8; 95% CI: 0.8, 3.8), older adolescents (AOR 1.4; 95% CI: 0.7, 3.1) and urban adolescents (AOR 10.5; 95% CI: 3.9, 28.2) were at higher risk of Vitamin D deficiency [70].

Two papers investigated the prevalence of iodine deficiency; it was 1.88% for severe deficiency (<20μg/L), 25.2% for moderate deficiency (50–99.9 μg/L) and 20.5% for mild deficiency (20–49.9 μg/L), and excess iodine (>300 μg/L) was found in 12.2%, in the age group12-14 years. The prevalence of goitre was 48.9% (girls 65%, boys 35%). Factors include female sex (AOR = 3.5; 95% CI: 2.6–4.9), living in a temperate climate (AOR = 0.6; 95% CI: 0.4–0.9), and a low frequency of iodized salt use (AOR = 0.5; 95% CI: 0.3–0.7) (67). The magnitude of serum folate deficiency (<6.8nmol/L) was 14.7% [69] (Table 2).

**Table 2:** Studies focusing on micronutrients.

Authors (y)	Objective (s)	Study design	Settings	Sample	Age	Sex	Exposure (s)	Outcomes (s)	Main findings
Desalegn D, 2014 [74]	To determine the prevalence, severity, and predictors of nutritional IDA	Cross sectional study	Urban	586 (269 aged 10–12)	10–12	M/F	-	Iron deficiency anemia (Hb <12 g/dl)	Iron deficiency anemia 32.7%
Wakao T, *2015* [70]	To determined vitamin D deficiency and its predictors	Cross sectional study	Urban/rural	174	11–18	M/F	Age, sex, parental education, wealth index	vitamin D deficiency (25(OH)D <50 nmol/L)	Vitamin D deficiency was 42% (girls 51.5%, boys 29.3%). Females (AOR = 1.76; 95% CI: 0.8, 3.8), older adolescent (AOR = 1.4; 95% CI: 0.7, 3.1) and urban residence (AOR = 10.5; 95% CI: 3.9, 28.2) are at higher risk of Vitamin D deficiency.
DHS report, 2016 [75]	To assess adolescent nutrition, including anaemia	Repeated cross-sectional survey	Urban/rural	-	15–19	M/F	Sex and setting	Anemia ((Hb <12 g/dl)	Anemia: urban girls 16.7%, rural girls 20.4%, Urban boys (8.6%), rural boys (20.4%). Anemia trends: in girls 15–19 years: 2005 (23.6; severe 1.0, moderate 12.7, mild 9.8), 2011 (13.3; severe 1.0, moderate 4.7%, mild 7.5%), 2016 (19.6; severe 0.9%, moderate 7.3%, mild 11.4%). Anemia trend: in boys 15–19 years: 2011: 17.7% (0.4% Severe, 2.4 moderate and 14.9 mild), 2016 18.2% (severe 0.1%, moderate 3.6%, and mild 14.4%).
Ministry of Health, 2016 [69]	Ethiopia National Micronutrient Survey to estimate the prevalence of selected micronutrient (Iron, Folate, vitamin A, Retinol, Zinc, Iodine, Vitamin B12) deficiencies	Cross-sectional study	Urban/rural	722	12–19 years	M/F	Age	Anemia, level of vitamin A, Vitamin B12, deficiencies of Zinc, iodine, folate	Anemia ((Hb <12 g/dL):14.9% (moderate (Hb 8–12 g/dL14.4%, severe (Hb<8g/dL 0.5%) in age of 12–14 years and 11.8% (10.5% moderate and 1.3% severe) in age 15–19 years. Iron deficiency (Ferritin<15) was 8.6%. Iron deficiency anemia (Ferritin) was 2.6% and (STFR) was 4.3% in age 12–14 years. Mean (SD) Vitamin A status was 1.20±0.35 and % (Retinol <0.7 μmol/l) was 6.3% in age 12–14 years. Iron deficiency (Ferritin<15) was 10.0%, (STFR<4.4) 83.8%, and IDA (Ferritin) 3.2%, IDA (serum transforming receptor) was 4.7%, vitamin A mean value 1.40±0.43, and % (Retinol <0.7 μmol/l) was 3.2% in age 15–19 years. Deficiencies: Zinc 38%, Iodine 1.88% for severe deficiency (<20μg/L), 25.2% for moderate deficiency (50–99.9 μg/L) and 20.5% for mild deficiency (20–49.9 μg/L). Excess iodine (>300 μg/L) 12.2% in age group 12–14 years. Deficiencies: Serum folate (<6.8nmol/L) 14.7%, Vitamin B12 (<203 pg/ml) 13.6%, iodine (Severe deficiency(<20 μg/L) 1.9%, moderate deficiency (50–99.9 μg/L) 28.6%, mild deficiency (20–49.9 μg/L) 21.9%, and Excess (>300 μg/L) 7.9% in age group 15–19 years.
Teji K, 2016 [39]	To assess the prevalence of anaemia and nutritional status of adolescent girls	Cross-sectional study	Urban/rural	547	10–19	F	-	Anemia ((Hb <12 g/dl)	Anemia 32% (HGB<12), severe 1.8% (HGB<7), moderate 3.8% (HGB 7–9.9), and mild 26.3% (HGB 10–11.9)
Getaneh Z, 2017 [68]	To assess the prevalence and associated factors of anemia	Cross-sectional	Urban	523 (332 aged 11–14)	11–14)	M/F	-	Anemia (Hb <12 g/dl)	Anemia 13.4%
Workie S, 2017 [67]	To assess the prevalence of iodine deficiency disorder	Cross-sectional study	Urban/rural	718	10–20+	M/F	Age, sex, setting, use of iodized salt	Iodine deficiency disorder (as measured by thyroid gland Enlargement)	Goiter 48.9% (boys 35.1%, girls 65.2%). Grade-1 goiter 36.9% and Grade-2 goiter 11.9%. Goiter is associated positively with girls (AOR = 3.5; 95%CI: 2.6–4.9) and negatively with regular use of iodized salt (AOR = 0.5; 95%CI: 0.3–0.7)
Gonete K, 2017 [71]	To assessed the prevalence and associated factors of anemia	Cross-sectional study	Urban/rural	462	15–19	F	Setting, dietary diversity, household food insecurity, source of water	Anemia (Hb <12 g/dl)	Overall anemia was 25.5%, (95% CI: 21.4, 29.2) with mild anemia 92.4%, moderate 5.9% and severe 1.7% Odds of having anemia among those with inadequate DDS was 2.1 higher (AOR = 2.1; 95% CI: 1.3, 3.5).
Wakayo T, 2018 [73]	To evaluate the association between Serum Vitamin D levels of 25(OH)D and handgrip strength	Cross-sectional study	Urban/rural	174	11–18	F	-	Serum Vitamin D level	Average serum 25(OH)D was 54.5 + 15.8 nmol/L.
Seyoum Y, 2019 [72]	To determine the prevalence of iron deficiency, low iron stores, and anemia and characterize selected risk factors	Cross-sectional study	Rural	257	15–19	F	-	Anemia (Hb <11 g/dl)	Anemia 8.7% (Hb <11 g/dL) and clinical iron deficiency 8.7% (Serum Ferretin <15 μg/L), but 41% had marginal iron stores (SF <50 μg/L).
Mengistu G 2019 [77]	To assess the prevalence of anemia and associated factors	Cross sectional	Urban	423	10–19	**F**	Age, DDS, family zise, family income,	Iron deficiency anemia (Hb <12 g/dl)	Anemia 11.1%. Predictors are: family size>5 *[AOR = 3*.*2*, *95*%*CI*: *1*.*3–7*.*9)*, lower average family income (*AOR = 10; 95*%*CI; 2*.*5–41*.*3)*.
Demelash S 2019 [78]	To assess the prevalence of anemia and its associated factors	Cross-sectional study	Urban	594	15–19	**F**	-	Anemia (Hb<12 g/dl)	Anemia prevalence 21.1% (CI: 17.4, 24).
Gebreyesus SH 2019 [79]	To evaluate the prevalence of anaemia	Cross sectional study	Urban/rural	1323	10–19	**F**	Age, residency, food insecurity	Anemia (Hb< 12.0 g/dl)	Anemia 28.8% (Urban 31.6%, rural19%) Anemia predictors: Younger (10–14 years) adolescent (AOR = 2.0; 95% CI: 1.1, 3.8)
Regasa RT, 2019 [80]	To determine the status of anemia and its anthropometric, dietary and socio demographic determinants	Cross-sectional study	Urban/rural	448	10–19	**F**	Age, residency, family size, parental education family wealth status, DDS,	Anemia (Hb< 12.0 g/dl, mild (10–11.9 g\dl), moderate (7.0–9.9 g/dl,) or severe (<7 g\dl)	Anemia 27% (95% CI: 22.9–31%, mild 23% and moderate 4%) Associated factors: age (younger 10–14 years 38.6%, older age 15–19 years 12.6%, p-value <0.05), settings (rural 45.3%, urban 12.5%, p-value <0.05)
Gebremichael G, 2020 [76]	To investigate the prevalence of goiter and associated factors	Cross-sectional study	Urban/rural	576	10–19	**M/F**	Sex, age, family history of goiter, residence, use of iodized salt, DDS, altitude	Goiter	Goiter 42.5% (95% CI: 38.4%, 46.7%; boys 34%, girls 50.9%) Goiter predictors: Being female (AOR = 1.8; 95% CI: 1.2, 2.9), family history of goiter (AOR = 3.6; 95% CI: 2.3, 5.7), lack of meat consumption (AOR = 2.5; 95% CI: 1.2, 5.3), lack of milk consumption (AOR = 2.2; 95% CI: 1.2, 4.0), and inadequate use of iodized salt (AOR = 7.1; 95% CI: 3.8, 12.9)

#### Diet diversity

Nine studies [28, 44, 72, 80–85] on a sample of 6 112 adolescents collected dietary data through food frequency questionnaires or 24 hour recalls. Three of these included both girls and boys [28, 44, 81], and the rest included only girls. Six included adolescents from both urban and rural [28, 80–83, 85] settings, two were from urban settings [44, 84] and one was from a rural setting [72]. The lowest prevalence of adequate dietary diversity ((DDS ≥5) was 4.3%, reported in girls from rural areas of Arisi zone (Southeast Ethiopia) in Oromia region [72]. Moreover, a high prevalence of low DDS was reported in different settings: 85.3% in rural and 58.5% in urban adolescents in northwestern Ethiopia [81]. Only 17.5% of adolescents consumed animal source foods in this study [81]. Another study from urban and rural areas of northwestern Ethiopia reported a similar prevalence of low DDS (85.5%) with food secure adolescents more likely to have an adequate DDS (AOR = 1.5, 95% CI 1.03, 2.1) compared to their food-insecure counterparts [82]. In urban settings of northwestern Ethiopia,75.4% (95% CI: 72.3, 78.6) of adolescents had an adequate DDS, higher among those from private schools (AOR = 3.2; 95% CI:1.9,5.3) and from merchant (well-off) families (AOR = 2.4; 95% CI: 1.1,5.5) [84].

Three studies reported mean DDS scores below the average recommended value; 3.3 in southwest Ethiopia [80], 3.5 in northern Ethiopia [83], and 4.3 in Jimma Town [85]. Only one study from an urban setting in south western Ethiopia (Jimma town) reported mean DDS above average (6.97) and cereal based (99.6%) and vegetables (73.9%) diet were the two commonly consumed food types. However, this study did not report the prevalence of low/high DDS [44].

In terms of site preference for nutrition interventions, as reported by adolescents, schools (45%), health centers (27%) and health posts (26%) were the preferred public facilities for provision of iron supplements to school adolescents, while schools (11%), health centers (47%) and health posts (41%) were the preferred public facilities for provision of iron supplements to out-of-school adolescents [83]. In the same study, it was indicated that a lack of nutrition messages specifically for young people, low community awareness about adolescent nutrition, religious and cultural influences, perceiving iron as a contraceptive than a nutrition product, and lack of confidence in the supplementation value of iron tablets are barriers to the uptake of adolescent nutrition interventions in northern Ethiopia (Table 3).

**Table 3:** Studies focusing on dietary diversity score (DDS).

Author (y)	Objective (s)	Study design	Settings	Sample	Age	Sex	Exposure	Outcomes	Main finding including description for each article
Herrador Z, 2015 [81]	To identify associated factors for low dietary diversity and lack of consumption of animal source food (ASF)	A cross-sectional survey with an additional follow up observation study	Urban/rural	886 (320 aged 10–18)	10–18	M/F	-	DDS	Low DDS; rural 85.3%, urban 58.5%. Consumption of animal source food 17.5%.
Mulugeta A, 2015 [83]	To examine means of reaching adolescent girls for iron supplementation	Cross-sectional study	Urban/rural	828	15–19	F	-	DDS	Prevalence of low, medium and high DDS was 54%, 42.9% and 3.1%, respectively. Mean DDS: 3.5 Schools (45%), health centers (27%) and health posts (26%) were the preferred public facilities for provision of iron supplements to school adolescents. Schools (11%), health centers (47%) and health posts (41%) were the preferred public facilities for provision of iron supplements to out of school adolescent
Tamiru D, 2016 [28]	To assess the effectiveness of school-based health and nutrition Intervention, supported with backyard gardening, on dietary diversity	Quasi experimental study	Urban/rural	1000	10–19	**M/F**	School-based health and nutrition intervention supported with backyard gardening	DDS	Prevalence of adequate DDS in the intervention group across time was 34.8% at baseline, 65.6% at midline and 74.7% at end line. Prevalence of adequate DDS in the control group across time was (32.1%) at baseline, (49.4%) at midline and (48.8%) end line. Effect of the intervention between intervention and control group: there was statistically significant difference at mid-point (F = 5.64, p = 0.042) and end (F = 5.85, p b 0.001).
Melaku Y 2017 [85]	To assess optimal dietary practices and nutritional knowledge	Cross sectional study	Urban/rural	455	14–19	**F**	Age, residency, maternal education, father occupation, family size	DDS	Mean (±SD) DDS was 4.3 ± 1.4. Low (<5) DDS 61.3% (Urban 37.7%, rural 50.0%) Low DDS predictors: attending government school (AOR = 5.2; 95% CI: 2.9,9.4), mothers illiterate (AOR = 7.7; 95%CI:3.4, 17.2), PRIMARY level education (AOR = 5.4; 95%CI: 2.6, 11.3), lower family economic status (AOR = 1.9; 95%CI: 1.0, 3.4)
Gali N, 2017 [44]	To assess emerging nutritional problems and their association with dietary intake among school adolescents	Cross-sectional study	Urban	546	Mean age 15.4 (SD 1.9)	M/F	-	DDS	Mean DDS was 6.97±1.15. Cereal based diets (99.6%) and vegetables (73.9%) were the two most common foods types consumed by adolescents.
Birru SM, 2018 [84]	To assess the dietary diversity of school adolescent girls in the context of urban Northwest Ethiopia	Cross-sectional study	Urban	768	10–19	F	School type, family occupation	DDS	Adequate DDS 75.4% (95%CI (72.3, 78.6). Adequate DDS associated with attending private school (AOR = 3.2; 95%CI: 1.9,5.3), being from merchant family (AOR = 2.4; 95%CI: 1.1,5.5)
Seyoum Y, 2019 [72]	To assess the prevalence of adequate dietary diversity among adolescents	Cross-sectional study	Rural	257	15–19	F	-	DDS	Only 4.3% of the adolescent girls had adequate dietary diversity (WDDS ≥5)
Tariku A, 2019 [82]	To assess the prevalence and associated factors of dietary diversity in adolescent girls	Cross-sectional study	Urban/rural	1550	10–19	F	food insecurity	DDS	Adequate dietary diversity was 14.5 (95% CI 12.9, 16.2), Households food security 74.9% Food secure adolescent are more likely to have adequate DDS (AOR = 1.5, 95% CI 1.03, 2.1)
Regasa RT 2019 [80]	To determine the dietary diversity of adolescents	Cross sectional study	Urban/rural	448	10–19	F	-	DDS	Mean DDS 3.3 + 1.2 Low DDS 56%, moderate DDS 41% and high DDS 3%

#### Food insecurity

Seven studies [23–27, 46, 86] with a sample size of 10 866 adolescents assessed food insecurity, of which five came from the Jimma Longitudinal Family Survey of Youth (JLFSY) study [23–27] which followed 2 084 adolescents over three years. The remaining two studies were cross sectional surveys with a sample size of 784 adolescents in areas producing Khat (a common evergreen plant in eastern Africa used for its psychoactive properties) and coffee. Food insecurity was assessed using the adolescent food insecurity assessment scale adopted from household food security questionnaire [107], which enquires about their experience or concern about access to food or money.

The prevalence of food insecurity in Jimma zone (urban and rural settings) was 59.6% [86]. In this study, female adolescents (AOR = 2.2, 95%CI:1.4, 3.5), household food insecurity (AOR = 9.4,95%CI:5.5, 16.2), a male head of household (AOR = 2.8, 95% CI:1.4, 5.3), a high dependency ratio (AOR = 2.5, 95% CI: 1.5, 4.5), a household head with no formal education (AOR = 4.9, 95% CI: 2.6,9.2) and a family which does not own farming land (AOR = 2.5, 95% CI: 1.2, 5.0) were positively associated with food insecurity [86]. The prevalence of food insecurity in Khat and Coffee producing areas of Sidama zone was lower at 38.0% (boys 40.2% and girls 35.9%; p-value 0.412) [46]. The prevalence of food insecurity was higher in coffee (43%) compared to khat producing areas (32.4%) [46].

The Jimma Longitudinal Family Survey of Youth (JLFSY) study was started in 2005 and has 3-yearly follow-ups [23–27]. In this longitudinal cohort, 20.4%, 48.4% and 20.6% of adolescents were food insecure during each consecutive round of the survey respectively [27]. In addition, 5.5% girls and 4.4% boys (P = 0.331) were from food insecure households in all three follow ups [25]. The mean height of food insecure girls was shorter by 0.87 cm (P<0.001) compared with food secure girls at baseline [25]. Predictors of food insecurity in the longitudinal study include, urban households within low (AOR = 1.7; 95% CI: 1.2, 2.5) and middle (AOR = 1.8; 95% CI: 1.2, 2.6) compared to high income tertiles were nearly twice as likely to suffer from chronic food insecurity [24]. Female sex (AOR = 1.6; 95% CI: 1.2, 2.1), high dependency ratio (AOR = 1.5; 95% CI: 1.0, 2.2) and household food insecurity (AOR = 2.7; 95% CI: 2.0, 3.6) among adolescents in urban, semi-urban, and rural areas were positively associated with food insecurity, while higher educational status was negatively associated (AOR = 0.5; 95% CI: 0.3, 0.8) [24]. Food insecure adolescents had lower DDS (P = 0.001), low mean food variety score (P = 0.001) and a lower frequency of consuming animal source foods (P = 0.001) compared to food secure adolescents [23] (Table 4).

**Table 4:** Studies focusing on food insecurity.

First author, y	Main objective (s)	Study design	Setting: Rural/urban	Sample size	Age (y)	Sex	Exposure (s)	Outcome (s)	Main findings
Hadley C, 2008 [26]	To examine the relationship between household and individual level food insecurity and health status among adolescent boys and girls	Data from the Jimma population based Longitudinal Family Survey of Youth (JLFSY) using multi-stage stratified cluster sampling method	Urban/rural	2084	13–17	M/F	Sex	Food insecurity assessed by a 6-item household food insecurity scale	Overall food insecure adolescents 13.9% (adolescents from medium food insecure households 30.8% (Boys 12.4%, girls 15.3%) and from severe food insecure household 20.5% (boys 20.9%, girls 41.0%) Boys and girls were equally likely to be living in severely food insecure households. Despite no differences in their households’ food insecurity status, girls were more likely than boys to report being food insecure themselves
Belachew T, 2012 [24]	To identify predictors of food insecurity among adolescents	Data from the Jimma Longitudinal Family Survey of Youth (JLFSY)	Urban/rural	1911	13–17	M/F	Residence,	Chronic Food insecurity	20.5% of adolescents were food insecure in the first-round survey and increased to 48.4% one year later. In the one year follow up 54.8% and 14.0% of the youth encountered transient and chronic food insecurity respectively. In urban households with low (AOR = 1.7; 95% CI: 1.2, 2.5) and middle (AOR = 1.8, 95% CI: 1.2, 2.6) income tertiles were nearly twice as likely to suffer from chronic food insecurity. Female sex (AOR = 1.6, 95% CI: 1.2, 2.1), high dependency ratio (AOR = 1.5; 95% CI: 1.0, 2.2) and household food insecurity (AOR = 2.7; 95% CI: 2.0, 3.6) were predictors of chronic adolescent food insecurity in urban, semi-urban, and rural areas, educational status of the adolescents was negatively associated with chronic food insecurity (AOR = 0.5; 95% CI: 0.3, 0.8)
Belachew T, 2013 [25]	To examine the association between food insecurity and linear growth among adolescents	The Jimma Longitudinal Family Survey of Youth (JLFSY)	Urban/rural	2084	13–17	M/F	Sex	Food insecurity	Food insecurity: at baseline and 1 year follow up was 15.9% in girls and 12.2% in boys (P = 0.018). In all the 3 follow-ups 5.5% girls and 4.4% boys (P = 0.331) were from food insecure households. Girls (40%) and boys (36.6%) (P = 0.045) were food insecure at least in one of the three survey rounds. Trends of food insecurity increased from 20.5% at the baseline to 48.4% on the 1 year follow up, and reversed down to 27.1% at the 2 years follow up survey. The mean height of food insecure girls was shorter by 0.87 cm (P<0.001) compared with food secure girls at baseline. But, at the follow up period, the heights of food insecure girls increased by 0.38 cm more per year compared with food secure girls (P<0.066). For boys, no significant difference in the mean height between food insecure and secured boys at baseline as well as over the follow up period.
Belachew T, 2013 [23]	To determine the association between adolescent food insecurity and dietary practices	Data from the first round survey the Jimma Longitudinal Family Survey of Youth (JLFSY)	Urban/rural	2084	13–17	M/F	Food insecurity	Dietary practice	Transient food insecure adolescents 20.5%. Coping to food insecurity: reducing daily food frequency (89.3%), worrying about running out of food (81.8%), spending the whole day without eating (23.8%) and asking for food or money to buy food/begging (20.8%). Food insecure adolescents had low dietary diversity score (P,0.001), low mean food variety score (P,0.001) and low frequency of consuming animal source foods (P,0.001).
Mulusew G, 2017 [27]	To examine the effect of food insecurity on self-rated health status	The Jimma Longitudinal Family Survey of Youth (JLFSY)	Urban/rural	1,919	14–22	M/F	-	Food insecurity assessed by 4-item adolescent food insecurity scale	20.4%, 48.4% and 20.6% of adolescents were food insecure during each consecutive round of the survey respectively. Adolescents with food insecurity were associated with self-rated health status (β = 0.28, P < 0.001)
Juju DB, 2018 [46]	To assess food security of adolescents in the selected khat and coffee-growing areas	Cross-sectional study	Rural	234	12–18	M/F	Sex	Food insecurity experiences	Adolescent with food insecurity 38.0% (boys 40.2% and girls 35.9%; p-value 0.412).
Gizaw G, 2018 [86]	To assess the prevalence and factors associated with adolescent food insecurity among coffee producing districts of Jimma Zone	Cross-sectional study	Urban/rural	550	10–19	M/F	Sex, household food insecurity, dependency ration, household head education, owner of farming land, wealth index	Adolescent food insecurity	Food insecure adolescents 59.6%. Female adolescents (AOR = 2.2; 95% CI: 1.4–3.5), household food insecurity (AOR = 9.4; 95% CI: 5.5–16.2), male of household heads (AOR = 2.8; 95% CI: 1.4–5.3), high dependency ratio (AOR = 2.5; 95% CI: 1.5–4.5), not formally educated household head (AOR = 4.9; 95% CI: 2.6–9.2) and have no own land for farm (AOR = 2.5; 95% CI: 1.2–4.9) were positively independent predictors of adolescent food insecurity.

### Eating disorders

Two studies [87, 88], both from Addis Ababa, assessed disordered eating and unhealthy weight control behaviors in adolescents [87, 88]. The prevalence of eating disorders was 8.6% (95% CI 4.9, 12.3) [87]. Female sex (AOR = 1.8; 95% CI: 1.0, 3.0) and being from less educated mother predicted a higher risk of eating disorders. Compared with no maternal schooling, maternal primary level education was associated with an AOR of 0.3 (95% CI: 0.1, 0.8), certificate/diploma with an AOR of 0.2 (95% CI: 0.1, 0.6) and a university degree or above with an AOR of 0.2 (95% CI: 0.1, 0.4) [87]. The prevalence of unhealthy weight control behavior was 31%, specifically purging behavior was 1.5% and non-purging weight control behavior was 30% [88]. In this study, predictors of unhealthy weight control behavior were being adolescent from a wealthier family (medium wealth index: AOR = 1.99; 95% CI:1.15, 3.45) and higher wealth index: AOR = 2.07; 95% CI: 1.30, 2.8), high perceived body weight (AOR = 3.01; 95% CI: 1.11, 8.11), higher BMI/overweight (AOR = 3.28; 95% CI:1.54, 7.01), and adolescent with severe depression (AOR = 4.09; 95% CI: 1.73,9.96) [88].

## Discussion

In this review, it was possible to extract, synthesize and summarize considerable data on nutritional status and associated factors, food insecurity, dietary diversity, micronutrient status, and disordered eating from studies among adolescents in Ethiopia. The review generally showed that there is more undernutrition (stunting, thinness and micronutrient deficiencies) than overweight among adolescents. The prevalence of thinness and stunting is higher among boys and rural adolescents whereas overweight and obesity are higher among girls and urban adolescents. The review also revealed that adolescent food insecurity and low dietary diversity are common. Consequently, a large proportion of adolescents have one or more micronutrient deficiencies. About 80% and 60% of adolescents from rural and urban settings respectively were found to have low dietary diversity. Our review supports a report from WHO [108] which documented that the magnitude of undernutrition, micronutrient deficiency, over-nutrition, inadequate or unhealthy diet and life styles is high among adolescents in LMICs. The finding from the current review showed that the magnitude of undernutrition and low DDS is substantial. Although the prevalence of overweight is low compared to that of undernutrition, it appears that problems of overnutrition are emerging before Ethiopia has dealt with the burden of under-nutrition. This is in line with global data which shows that a double burden of malnutrition is increasing in LMICs [109] as they experience rapid economic growth, urbanization, and changes in dietary habits and levels of physical activity.

Undernutrition (underweight, stunting and thinness) is more prevalent in younger adolescents, boys, and rural adolescents, whereas overnutrition (overweight and obesity) is higher in females and urban adolescents. Adolescents in rural settings are more likely to be engaged in various labour intensive (energy consuming) domestic activities to support their family. In addition, household food insecurity is higher in rural compared to urban communities because of low literacy rates, recurrent droughts, and lack of diversity in sources of income. In contrast, because of urbanization and concomitant changes in lifestyle, urban adolescents are more likely to consume low quality foods such as sweets and fast foods, have more screen time and spend more time sedentary. There are more limited opportunities for physical activity in urban environments, especially for girls, because of overcrowding and lack of space. In a recent qualitative study, we identified that boys have more opportunity for leisure time and outdoor physical activity than girls [110].

Geographically, undernutrition is higher in northern compared to southern Ethiopia. The community in the north Ethiopia is characterized by subsistence farming where crops are the main source of income, there is greater food insecurity, and nutritional habits and experience are greatly influenced by cultural values [111] such as fasting (no animal-source meals for the majority of months of the year) [112]. In contrast, the southern region of the country is known for highly-productive horticulture of fruits and vegetables in addition to other crops, which are easily accessible to the local community.

Trends in the nutritional status of adolescents over the study period showed no clear secular trends. This could happen for the fact that the reviewed studies covered quite a limited time period, and importantly were not truly longitudinal (they represent separate studies in different populations) and are therefore not ideal for a trend analysis. The prevalence of undernutrition and overnutrition has changed little, and both have coexisted in the community over the last decade. This could happen because, despite rapid economic growth and urbanization, wide wealth disparity persists in Ethiopia. The United Nations have adopted the first ever UN Decade of Action on Nutrition, from 2016–2025 to realize the goal set to eliminate all forms of malnutrition by 2030 [113]. To date, several of the nutrition targets which were agreed upon remain unmet and on the contrary, the double burden of malnutrition challenge is increasing. It is predicted that, if current trends continue, the absolute number of overweight people will have increased from almost 2 billion today, to 3.3 billion by 2030, equal to one third of the projected world population [114]. Nutrition interventions for the current generation of adolescents in Ethiopia would require context- and community-specific intervention approaches to address all forms of malnutrition.

Micronutrient deficiencies are also common in adolescents, with deficiencies of iron, zinc, iodine, folic acid, and vitamins A and B12 being the most common. Factors that could contribute are a lack of dietary diversity, a lack of fortified foods, food insecurity and low general knowledge and awareness about the need for micronutrients for health. While there was a steady reduction in iron deficiency anemia in girls between 2000 and 2016, there is an increase in boys over the same time period. This can be explained by the targeting of national initiatives selectively towards women of reproductive age over recent decades. Despite the high burden, there are no national or regional initiatives to tackle micronutrient deficiencies in the adolescent population at ground level.

Risk factors for undernutrition identified in this review include low socioeconomic status, maternal education and dietary diversity, food insecurity, higher family size, attending a public school, younger age, male sex and living in a rural setting. Risk factors for overnutrition included female sex, urban settings, lower levels of physical activity or more sedentary lifestyles, and coming from more wealthy families, having access to sweets/fast foods, older age and attending private schools. The sociodemographic and economic factors are modifiable causes of malnutrition, which could be addressed through effective context-relevant interventions, designed with the involvement of policy makers, experts, adolescents and their families.

The impact from the double burden of malnutrition could occur at the level of individual, household or nation. Individuals who were under-nourished as infants can have increased weight gain and obesity during adolescence or late in adulthood, while it is also possible for an obese person to have micronutrient deficiencies concomitantly. In the same household, some family members may be under-nourished while others are obese. The situation is the same for a given country.

Effective intervention strategies are required to tackle the double burden of malnutrition emerging in Ethiopia. The national strategy for adolescent and youth health and nutrition [115], produced by the ministry of health, recommends promoting participation and leadership by adolescents in the planning and implementations of adolescent-related nutrition programmes, implementing innovative health education and prevention programmes using the health extension programmes, schools, mass media and digital technologies. Specifically recommended interventions [115] include improving consumption of a balanced diet, with an emphasis on locally available and iron-rich foods, promoting healthy dietary habits, creating awareness of the intergenerational effects of malnutrition, creating community awareness on gender bias in household food distribution, targeted supplementation of iron and folic acid, the scaling up of facility-based nutrition assessment and counselling programs, advocacy and promotion of food fortification. These recommendations are in line with the WHO guide for implementation of effective action for improve adolescent nutrition [116]. These efforts will be more effective if global co-ordination, collaboration and integration can be achieved.

As adolescents are open for new ideas, and are concerned and interested about their health and life perspective, they could serve as the agents for change. Adolescence is therefore a window of opportunity for intervention [11, 117]. Habits and experiences built during adolescence are more likely to last throughout life to some extent. Engaging adolescents in the design of their own nutrition and health interventions is likely to influence them positively. Involving young people as educators and intervention providers enables them to take responsibility for their nutritional health and is a way of allowing research to reach wider and hard-to-reach communities. A comprehensive intervention model that considers health, nutrition and wellbeing in general is more acceptable and impactful than targeting a single problem [116]. Such intervention models could combine counseling for nutrition and wellbeing, family life education, life skill trainings and positive behavior promotion (rather than focusing on discouraging negative behavior) to empower young people [118].

### Strengths and limitations

Strengths of this review included a rigorous, standardised methodological approach and the involvement of multidisciplinary expertise through the TALENT collaboration. We have used definition of BMI for age z-score >1 for overweight and BMI for age z-score>2 for obesity in the meta-analysis for overnutrition. A limitation was that we were not able to use data for overweight when it was defined by weight for age z-score. Trend analysis overtime was not possible because of the limited range of years covered by the studies and the studies are mostly separate surveys in different populations rather than longitudinal data in the same population or setting.

## Conclusions

While the magnitude of undernutrition remains high in Ethiopia, overnutrition is an emerging problem, leading to a double burden of malnutrition. Stunting and thinness are higher in boys and in rural settings while overweight and obesity are higher in girls and in urban settings. Half of adolescents found to have at least one micronutrient deficiency. There is a paucity of evidence from intervention studies to improve adolescent health and nutrition in Ethiopia. Therefore, appropriate and context-relevant intervention studies that address the various forms of malnutrition among adolescents should be designed and implemented, preferably with the active participation of adolescents themselves.

## Supporting information

S1 Checklist (DOCX)

S1 Data (XLSX)

S2 Data (XLSX)

S3 Data (XLSX)

## References

[1] Frisch RE, Revelle R. Height and weight at menarche and a hypothesis of menarche. Arch Dis Child. 1971 Oct;46(249):695–701. doi: 10.1136/adc.46.249.695 5118059 PMC1647814

[2] Das JK, Salam RA, Thornburg KL, Prentice AM, Campisi S, Lassi ZS, et al. Nutrition in adolescents: physiology, metabolism, and nutritional needs: Adolescents: physiology, metabolism, and nutrition. Ann N Y Acad Sci. 2017 Apr;1393(1):21–33. 28436102 10.1111/nyas.13330

[3] Biesalski Hans K, Jana T. Micronutrients in the life cycle: Requirements and sufficient supply. NFS J. 2018 Jun;11:1–11.

[4] Caskey MM, Jr VAA. Research Summary: Young Adolescents’ Developmental Characteristics.: 9.

[5] UNICEF. For every child, every right the Convention on the Rights of the Child at a crossroads [Internet]. New York: United Nations Children’s Fund; 2019 [cited 2020 Aug 15]. Available from: https://www.unicef.org/reports/convention-rights-child-crossroads-2019

[6] Central Statistical Agency [Ethiopia]. 2014. Ethiopia Mini Demographic and Health Survey 2014. Addis Ababa, Ethiopia.

[7] Wrottesley SV, Mates E, Brennan E, Bijalwan V, Menezes R, Ray S, et al. Nutritional status of school-age children and adolescents in low- and middle-income countries across seven global regions: a synthesis of scoping reviews. Public Health Nutr. 2022 Feb 14;1–33. doi: 10.1017/S1368980022000350 35156607 PMC11077463

[8] Food and Agriculture Organization of the United Nations(FAO). The double burden of malnutrition Case studies from six developing countries FOOD AND NUTRITION. 2006. 19172986

[9] Shrimpton R, Rokx C. The double burden of malnutrition: a review of global evidence.Health, Nutrition and Population (HNP) Discussion Paper. 2012.

[10] Calkins K, Devaskar SU. Fetal Origins of Adult Disease. Curr Probl Pediatr Adolesc Health Care. 2011 Jul;41(6):158–76. doi: 10.1016/j.cppeds.2011.01.001 21684471 PMC4608552

[11] Prentice AM, Ward KA, Goldberg GR, Jarjou LM, Moore SE, Fulford AJ, et al. Critical windows for nutritional interventions against stunting. Am J Clin Nutr. 2013 May 1;97(5):911–8. doi: 10.3945/ajcn.112.052332 23553163 PMC3628381

[12] World Health Organization. Global nutrition policy review: what does it take to scale up nutrition action? Geneva: World Health Organization; 2013.

[13] Ethiopia Approves National Food and Nutrition Policy _ Ethiopia _ Save the Children: Avilable at: https://ethiopia.savethechildren.net/news/ethiopia-approves-national-food-and-nutrition-policy.

[14] National Food and Nutrition Policy: Federal Democratic Republic Of Ethiopia, 2018.

[15] Reducing stunting in children: equity considerations for achieving the Global Nutrition Targets 2025. Geneva: World Health Organization; 2018. Licence: CC BY-NC-SA 3.0 IGO.

[16] Arksey H, O’Malley L. Scoping studies: towards a methodological framework. Int J Soc Res Methodol. 2005 Feb;8(1):19–32.

[17] Peters MDJ, Godfrey CM, Khalil H, McInerney P, Parker D, Soares CB. Guidance for conducting systematic scoping reviews: Int J Evid Based Healthc. 2015 Sep;13(3):141–6. doi: 10.1097/XEB.0000000000000050 26134548

[18] Hardy-Johnson Polly and Dhuria Preeti, Strommer Sofia, Weller Susie, Barker Mary, Fall Caroline, et al. Exploring the Diet and Physical Activity Behaviours of Adolescents Living in India and sub-Saharan Africa: A Qualitative Synthesis. Submitted to Public Health Nutrition. 10.1017/S1368980021002408 PMC10201354 34196267

[19] Moher D, Liberati A, Tetzlaff J, Altman DG, for the PRISMA Group. Preferred reporting items for systematic reviews and meta-analyses: the PRISMA statement. BMJ. 2009 Jul 21;339(jul21 1):b2535–b2535. 19622551 10.1136/bmj.b2535 PMC2714657

[20] Moola S, Munn Z, Tufanaru C, Aromataris E, Sears K, Sfetc R, et al. Chapter 7: Systematic Reviews of Etiology and Risk. In: Aromataris E, Munn Z, editors. JBI Manual for Evidence Synthesis [Internet]. JBI; 2020 [cited 2020 Sep 19]. Available from: https://wiki.jbi.global/display/MANUAL/Chapter+7%3A+Systematic+reviews+of+etiology+and+risk

[21] Tufanaru C, Munn Z, Aromataris E, Campbell J, Hopp L. Chapter 3: Systematic Reviews of Effectiveness. In: Aromataris E, Munn Z, editors. JBI Manual for Evidence Synthesis [Internet]. JBI; 2020 [cited 2020 Sep 19]. Available from: https://wiki.jbi.global/display/MANUAL/Chapter+3%3A+Systematic+reviews+of+effectiveness

[22] Assefa H, Belachew T, Negash L. Socio-demographic factors associated with underweight and stunting among adolescents in Ethiopia. Pan Afr Med J [Internet]. 2015 [cited 2020 Aug 15];20. Available from: http://www.panafrican-med-journal.com/content/article/20/252/full/ doi: 10.11604/pamj.2015.20.252.3588 26161175 PMC4484194

[23] Belachew T, Lindstrom D, Gebremariam A, Hogan D, Lachat C, Huybregts L, et al. Insecurity Food, Food Based Coping Strategies and Suboptimal Dietary Practices of Adolescents in Jimma Zone Southwest Ethiopia. Cameron DW, editor. PLoS ONE. 2013 Mar 12;8(3):e57643. 23554864 10.1371/journal.pone.0057643 PMC3595236

[24] Belachew T, Lindstrom D, Gebremariam A, Jira C, Hattori MK, Lachat C, et al. Predictors of chronic food insecurity among adolescents in Southwest Ethiopia: a longitudinal study. BMC Public Health. 2012 Dec;12(1):604. doi: 10.1186/1471-2458-12-604 22863140 PMC3439274

[25] Belachew T, Lindstrom D, Hadley C, Gebremariam A, Kasahun W, Kolsteren P. Food insecurity and linear growth of adolescents in Jimma Zone, Southwest Ethiopia. Nutr J. 2013 Dec;12(1):55. doi: 10.1186/1475-2891-12-55 23634785 PMC3671154

[26] Hadley C, Lindstrom D, Tessema F, Belachew T. Gender bias in the food insecurity experience of Ethiopian adolescents. Soc Sci Med. 2008 Jan;66(2):427–38. doi: 10.1016/j.socscimed.2007.08.025 17931763 PMC2791354

[27] Jebena MG, Lindstrom D, Lachat C, Belachew T, Kolsteren P. The effect of food insecurity on health status of adolescents in Ethiopia: longitudinal study. BMC Public Health. 2017 Dec;17(1):465. doi: 10.1186/s12889-017-4406-5 28521757 PMC5437384

[28] Tamiru D, Argaw A, Gerbaba M, Nigussie A, Ayana G, Belachew T. Improving dietary diversity of school adolescents through school based nutrition education and home gardening in Jimma Zone: Quasi-experimental design. Eat Behav. 2016;23:180–6. doi: 10.1016/j.eatbeh.2016.10.009 27816856

[29] Alemayehu T, Haidar J, Habte D. Adolescents’ undernutrition and its determinants among in-school communities of Ambo town, West Oromia, Ethiopia. East Afr J Public Health. 2010 Sep;7(3):263–7. doi: 10.4314/eajph.v7i3.64738 21516966

[30] Herrador Z, Sordo L, Gadisa E, Moreno J, Nieto J, Benito A, et al. Cross-Sectional Study of Malnutrition and Associated Factors among School Aged Children in Rural and Urban Settings of Fogera and Libo Kemkem Districts, Ethiopia. Jaspan HB, editor. PLoS ONE. 2014 Sep 29;9(9):e105880. doi: 10.1371/journal.pone.0105880 25265481 PMC4179248

[31] Gebreyohannes Y. Nutritional Status of Adolescents in Selected Government and Private Secondary Schools of Addis Ababa, Ethiopia. Int J Nutr Food Sci. 2014;3(6):504.

[32] Berheto TM, Mikitie WK, Argaw A. Urban-rural disparities in the nutritional status of school adolescent girls in the Mizan district, south-western Ethiopia. Rural Remote Health. 2015 Sep;15(3):3012. 26235698

[33] Melaku YA, Zello GA, Gill TK, Adams RJ, Shi Z. Prevalence and factors associated with stunting and thinness among adolescent students in Northern Ethiopia: a comparison to World Health Organization standards. Arch Public Health. 2015 Dec;73(1):44. 26516456 10.1186/s13690-015-0093-9 PMC4624644

[34] Wassie MM, Gete AA, Yesuf ME, Alene GD, Belay A, Moges T. Predictors of nutritional status of Ethiopian adolescent girls: a community based cross sectional study. BMC Nutr. 2015 Dec;1(1):20.

[35] Alelign T, Degarege A, Erko B. Prevalence and factors associated with undernutrition and anaemia among school children in Durbete Town, northwest Ethiopia. Arch Public Health. 2015 Dec;73(1):34. doi: 10.1186/s13690-015-0084-x 26261719 PMC4530480

[36] Weres ZG, Yebyo HG, Miruts KB, Gesesew HA, Woldehymanot TE (2015) Assessment of Adolescents’ Under Nutrition Level among School Students in Eastern Tigray, Ethiopia: A Cross-Sectional Study. J Nutr Food Sci 5: 402. doi: 10.4172/2155-9600.1000402

[37] Gebregyorgis T, Tadesse T, Atenafu A. Prevalence of Thinness and Stunting and Associated Factors among Adolescent School Girls in Adwa Town, North Ethiopia. Int J Food Sci. 2016;2016:1–8. doi: 10.1155/2016/8323982 27294107 PMC4884871

[38] Awel AA, Lema TB, Hebo HJ. Nutritional status and associated factors among primary school adolescents of pastoral and agro- pastoral communities, Mieso Woreda, Somali Region, Ethiopia: A comparative cross-sectional study. 2016;14.

[39] Teji K, Dessie Y, Assebe T, Abdo M. Anaemia and nutritional status of adolescent girls in Babile District, Eastern Ethiopia. Pan Afr Med J. 2016;24:62. doi: 10.11604/pamj.2016.24.62.6949 27642403 PMC5012790

[40] Roba K, M A. Nutritional Status and Its Associated Factors among School Adolescent Girls in Adama City, Central Ethiopia. J Nutr Food Sci [Internet]. 2016 [cited 2020 Aug 17];06(03). Available from: https://www.omicsonline.org/open-access/nutritional-status-and-its-associated-factors-among-school-adolescentgirls-in-adama-city-central-ethiopia-2155-9600-1000493.php?aid=73602

[41] Teferi DY, Atomssa GE, Mekonnen TC. Overweight and Undernutrition in the Cases of School-Going Adolescents in Wolaita Sodo Town, Southern Ethiopia: Cross-Sectional Study. J Nutr Metab. 2018;2018:1–10. doi: 10.1155/2018/8678561 29785306 PMC5896243

[42] Tegegne M, Sileshi S, Assefa T, Kalu A. Nutritional Status and Associated Factors of Adolescent School Girls, Goba Town, Southeast Ethiopia. 2016;9.

[43] Shegaze M. Magnitude and Determinants of Overweight and Obesity Among High School Adolescents in Addis Ababa, Ethiopia. J Food Nutr Sci. 2016;3(5):166.

[44] Gali N, Tamiru D, Tamrat M. The Emerging Nutritional Problems of School Adolescents: Overweight/Obesity and Associated Factors in Jimma Town, Ethiopia. J Pediatr Nurs. 2017 Jul;35:98–104. doi: 10.1016/j.pedn.2017.03.002 28728777

[45] Hassen K, Gizaw G, Belachew T. Dual Burden of Malnutrition Among Adolescents of Smallholder Coffee Farming Households of Jimma Zone, Southwest Ethiopia. Food Nutr Bull. 2017 Jun;38(2):196–208. doi: 10.1177/0379572117701660 28438035

[46] Juju D, Sekiyama M, Saito O. Food Security of Adolescents in Selected Khat- and Coffee-Growing Areas in the Sidama Zone, Southern Ethiopia. Nutrients. 2018 Jul 27;10(8):980. doi: 10.3390/nu10080980 30060510 PMC6115914

[47] Birru SM, Belew AK, Tariku A. One in three adolescent schoolgirls in urban northwest Ethiopia is stunted. Ital J Pediatr. 2018 Dec;44(1):32. doi: 10.1186/s13052-018-0459-z 29514659 PMC5842615

[48] Mekonnen T, Tariku A, Abebe SM. Overweight/obesity among school aged children in Bahir Dar City: cross sectional study. Ital J Pediatr. 2018 Dec;44(1):17. doi: 10.1186/s13052-018-0452-6 29361952 PMC5781282

[49] Tariku EZ, Abebe GA, Melketsedik ZA, Gutema BT. Prevalence and factors associated with stunting and thinness among school-age children in Arba Minch Health and Demographic Surveillance Site, Southern Ethiopia. Laar A, editor. PLOS ONE. 2018 Nov 2;13(11):e0206659. doi: 10.1371/journal.pone.0206659 30388149 PMC6214544

[50] Moges T, Gebremichael B, Shiferaw S, Yirgu R. Is inadequate play area in schools associated with overweight among students in Addis Ababa, Ethiopia? A comparative cross-sectional study. Epidemiol Health. 2018 May 12;40:e2018017. doi: 10.4178/epih.e2018017 29807411 PMC6060341

[51] Demilew YM, Emiru AA. Under nutrition and associated factors among school adolescents in Dangila Town, Northwest Ethiopia: a cross sectional study. Afr Health Sci. 2018 Aug 15;18(3):756. doi: 10.4314/ahs.v18i3.34 30603009 PMC6307005

[52] Mitiku H, Admassu D, Teklemariam Z, Weldegebreal F, Nigusse A. Nutritional status of school children in eastern Hararghe administrative zone, eastern Ethiopia. J Public Health. 2018 Feb;27(1):111–8.

[53] Girmay AM, Hassen MN (2018) Prevalence of Overweight and Obesity and Associated Factors among Private Primary School Students in Gulele Sub-City of Addis Ababa, Ethiopia. Epidemiology (Sunnyvale) 8: 352. doi: 10.4172/2161-1165.1000352

[54] Zenebe M, Gebremedhin S, Henry CJ, Regassa N. School feeding program has resulted in improved dietary diversity, nutritional status and class attendance of school children. Ital J Pediatr. 2018 Dec;44(1):16. doi: 10.1186/s13052-018-0449-1 29361948 PMC5782386

[55] Bidu KT, Hailemariam T, Negeri EL, Babure ZK. Prevalence and associated factors of undernutrition among school adolescents in Gobu Seyo District, East Wollega Zone, Oromia regional state of West Ethiopia, J. Publ. Health and Epidemiology, 2016; 10(7), pp. 251–269.

[56] Arage G, Assefa M, Worku T. Socio-demographic and economic factors are associated with nutritional status of adolescent school girls in Lay Guyint Woreda, Northwest Ethiopia. SAGE Open Med. 2019 Jan;7:205031211984467. doi: 10.1177/2050312119844679 31019699 PMC6469276

[57] Tariku A, Belew AK, Gonete KA, Hunegnaw MT, Muhammad EA, Demissie GD, et al. Stunting and Its Determinants among Adolescent Girls: Findings from the Nutrition Surveillance Project, Northwest Ethiopia. Ecol Food Nutr. 2019 Sep 3;58(5):481–94. doi: 10.1080/03670244.2019.1636793 31271301

[58] Belay E, Handebo S, Derso T, Tariku A, Sisay M. Prevalence and determinants of pre-adolescent (5–14 years) acute and chronic undernutrition in Lay Armachiho District, Ethiopia. Int J Equity Health. 2019 Dec;18(1):137. 31477149 10.1186/s12939-019-1041-z PMC6721279

[59] Zemene MA, Engidaw MT, Gebremariam AD, Asnakew DT, Tiruneh SA. Nutritional status and associated factors among high school adolescents in Debre Tabor Town, South Gondar Zone, Northcentral Ethiopia. BMC Nutr. 2019 Dec;5(1):43. doi: 10.1186/s40795-019-0306-7 32153956 PMC7050895

[60] Wolde T, Belachew T. Chronic undernutrition (stunting) is detrimental to academic performance among primary schools of adolescent children: a randomized cross sectional survey in Southern Ethiopia. BMC Res Notes. 2019 Dec;12(1):142. doi: 10.1186/s13104-019-4160-0 30876451 PMC6419846

[61] Daba DB, Shaweno T, Taye K, Workicho A. Magnitude of under nutrition and Associated Factors among Adolescent Street Children at Jimma Town, South West Ethiopia [Internet]. In Review; 2019 Aug [cited 2020 Aug 22]. Available from: https://www.researchsquare.com/article/rs-4257/v1

[62] Jikamo B, Samuel M. Does dietary diversity predict the nutritional status of adolescents in Jimma Zone, Southwest Ethiopia? BMC Res Notes. 2019 Dec;12(1):402. doi: 10.1186/s13104-019-4437-3 31307544 PMC6628467

[63] Beyene S. Anthropometric Assessment of Adolescent Nutritional Status in Two Drought-Prone Areas of Ethiopia. 2019;9(755):8.

[64] Berhe K, Gebremariam G. Magnitude and associated factors of undernutrition (underweight and stunting) among school adolescent girls in Hawzen Woreda (District), Tigray regional state, Northern Ethiopia: Cross-sectional study. BMC Res Notes. 2020 Dec;13(1):59. doi: 10.1186/s13104-020-4926-4 32029003 PMC7006198

[65] Kahssay M, Mohamed L, Gebre A. Nutritional Status of School Going Adolescent Girls in Awash Town, Afar Region, Ethiopia. J Environ Public Health. 2020 Feb 21;2020:1–9. doi: 10.1155/2020/7367139 32148529 PMC7054789

[66] Gagebo DD, Kerbo AA, Thangavel T. Undernutrition and Associated Factors among Adolescent Girls in Damot Sore District, Southern Ethiopia. J Nutr Metab. 2020 Jul 1;2020:1–11. doi: 10.1155/2020/5083140 32685206 PMC7350169

[67] Workie SB, Abebe YG, Gelaye AA, Mekonen TC. Assessing the status of iodine deficiency disorder (IDD) and associated factors in Wolaita and Dawro Zones School Adolescents, southern Ethiopia. BMC Res Notes. 2017 Dec;10(1):156. doi: 10.1186/s13104-017-2480-5 28420409 PMC5395884

[68] Getaneh Z, Enawgaw B, Engidaye G, Seyoum M, Berhane M, Abebe Z, et al. Prevalence of anemia and associated factors among school children in Gondar town public primary schools, northwest Ethiopia: A school-based cross-sectional study. van Wouwe JP, editor. PLOS ONE. 2017 Dec 28;12(12):e0190151. doi: 10.1371/journal.pone.0190151 29284032 PMC5746225

[69] Ethiopian National Micronutrient Survey Report, 2016, Ethiopian Public Health Institute, Ethiopia.

[70] Wakayo T, Belachew T, Vatanparast H, Whiting SJ. Vitamin D Deficiency and Its Predictors in a Country with Thirteen Months of Sunshine: The Case of School Children in Central Ethiopia. Carpenter DO, editor. PLOS ONE. 2015 Mar 30;10(3):e0120963. doi: 10.1371/journal.pone.0120963 25822900 PMC4387794

[71] Gonete KA, Tariku A, Wami SD, Derso T. Prevalence and associated factors of anemia among adolescent girls attending high schools in Dembia District, Northwest Ethiopia, 2017. Arch Public Health. 2018 Dec;76(1):79. doi: 10.1186/s13690-018-0324-y 30598822 PMC6302287

[72] Seyoum Y, Humblot C, Nicolas G, Thomas M, Baye K. Iron deficiency and anemia in adolescent girls consuming predominantly plant-based diets in rural Ethiopia. Sci Rep. 2019 Dec;9(1):17244. doi: 10.1038/s41598-019-53836-5 31754277 PMC6872871

[73] Wakayo T, Belachew T, Whiting SJ. Serum Vitamin D Level Associates With Handgrip Muscle Strength Among Ethiopian Schoolchildren: A Cross-Sectional Study. Food Nutr Bull. 2018 Mar;39(1):54–64. doi: 10.1177/0379572117724545 28823213

[74] Desalegn A, Mossie A, Gedefaw L. Nutritional Iron Deficiency Anemia: Magnitude and Its Predictors among School Age Children, Southwest Ethiopia: A Community Based Cross-Sectional Study. Schooling CM, editor. PLoS ONE. 2014 Dec 1;9(12):e114059. doi: 10.1371/journal.pone.0114059 25438147 PMC4250059

[75] Adolescent nutrition 2000–2017: Demographic and Health Survey (DHS) data on adolescents age 15–19. DHS comparative reports 47.

[76] Gebremichael G, Demena M, Egata G, Gebremichael B. Prevalence of Goiter and Associated Factors Among Adolescents in Gazgibla District, Northeast Ethiopia. Glob Adv Health Med. 2020 Jan;9:216495612092362. doi: 10.1177/2164956120923624 32435526 PMC7223861

[77] Mengistu G, Azage M, Gutema H. Iron Deficiency Anemia among In-School Adolescent Girls in Rural Area of Bahir Dar City Administration, North West Ethiopia. Anemia. 2019 Mar 21;2019:1–8. doi: 10.1155/2019/1097547 31016041 PMC6448345

[78] Demelash S, Murutse M. Prevalence of Anemia and its Associated Factors among School Adolescent Girls Addis Ababa, 2015. 2019;11.

[79] Gebreyesus SH, Endris BS, Beyene GT, Farah AM, Elias F, Bekele HN. Anaemia among adolescent girls in three districts in Ethiopia. BMC Public Health. 2019 Dec;19(1):92. doi: 10.1186/s12889-019-6422-0 30665390 PMC6341533

[80] Regasa RT, Haidar JA. Anemia and its determinant of in-school adolescent girls from rural Ethiopia: a school based cross-sectional study. BMC Womens Health. 2019 Dec;19(1):98. doi: 10.1186/s12905-019-0791-5 31315626 PMC6637513

[81] Herrador Z, Perez-Formigo J, Sordo L, Gadisa E, Moreno J, Benito A, et al. Low Dietary Diversity and Intake of Animal Source Foods among School Aged Children in Libo Kemkem and Fogera Districts, Ethiopia. van Wouwe J, editor. PLOS ONE. 2015 Jul 23;10(7):e0133435. doi: 10.1371/journal.pone.0133435 26203904 PMC4512702

[82] Tariku A, Gonete KA, Bikes GA, Alemu K, Belew AK, Wassie MM, et al. Household food insecurity predisposes to undiversified diet in northwest Ethiopia: finding from the baseline survey of nutrition project, 2016. BMC Res Notes. 2019 Dec;12(1):54. doi: 10.1186/s13104-019-4083-9 30678698 PMC6346507

[83] Mulugeta A, Tessema M, H/sellasie K, Seid O, Kidane G, Kebede A. Examining Means of Reaching Adolescent Girls for Iron Supplementation in Tigray, Northern Ethiopia. Nutrients. 2015 Nov 2;7(11):9033–45. doi: 10.3390/nu7115449 26540071 PMC4663577

[84] Birru SM, Tariku A, Belew AK. Improved dietary diversity of school adolescent girls in the context of urban Northwest Ethiopia: 2017. Ital J Pediatr. 2018 Dec;44(1):48. doi: 10.1186/s13052-018-0490-0 29695264 PMC5918967

[85] Melaku Y, Dirar A, Feyissa GT, Tamiru D. Optimal dietary practices and nutritional knowledge of school adolescent girls in Jimma Town, South West Ethiopia. Int J Adolesc Youth. 2018 Jul 3;23(3):299–307.

[86] Getu GH, Kalkidane HA. Intra- household food allocation among adolescents in coffee farming households in Jimma Zone, South west Ethiopia. J Geogr Reg Plan. 2018 Sep 30;11(9):134–42.

[87] Yirga B, Assefa Gelaw Y, Derso T, Wassie MM. Disordered eating attitude and associated factors among high school adolescents aged 12–19 years in Addis Ababa, Ethiopia: a cross-sectional study. BMC Res Notes. 2016 Dec;9(1):503. 27927224 10.1186/s13104-016-2318-6 PMC5143448

[88] Tuffa TA, Gebreyesus SH, Endris BS, Getnet Y, Abebe DS. Unhealthy weight control behaviors among Ethiopian female adolescents. Int J Eat Disord. 2020 Apr;53(4):525–32. doi: 10.1002/eat.23227 31944363

[89] Sisay BG, Haile D, Hassen HY, Gebreyesus SH. Performance of mid-upper arm circumference as a screening tool for identifying adolescents with overweight and obesity. Mota JF, editor. PLOS ONE. 2020 Jun 23;15(6):e0235063. doi: 10.1371/journal.pone.0235063 32574192 PMC7310830

[90] Roba AC, Gabriel-Micheal K, Zello GA, Jaffe J, Whiting SJ, Henry CJ. A Low Pulse Food Intake May Contribute to the Poor Nutritional Status and Low Dietary Intakes of Adolescent Girls in Rural Southern Ethiopia. Ecol Food Nutr. 2015 May 4;54(3):240–54. doi: 10.1080/03670244.2014.974593 25602600

[91] Gebregyorgis T, Tadesse T, Atenafu A. Prevalence of Thinness and Stunting and Associated Factors among Adolescent School Girls in Adwa Town, North Ethiopia. Int J Food Sci. 2016;2016:1–8. doi: 10.1155/2016/8323982 27294107 PMC4884871

[92] Zenebe M, Gebremedhin S, Henry CJ, Regassa N. School feeding program has resulted in improved dietary diversity, nutritional status and class attendance of school children. Ital J Pediatr. 2018 Dec;44(1):16. doi: 10.1186/s13052-018-0449-1 29361948 PMC5782386

[93] Taklual W, Baye S, Mekie M, Andualem T. Double Burden of Malnutrition among Female Adolescent Students in Bahir Dar City, Amhara, Ethiopia. BioMed Res Int. 2020 Aug 17;2020:1–10. doi: 10.1155/2020/6249524 32879884 PMC7448125

[94] Irenso AA, Dessie Y, Berhane Y, Assefa N, Canavan CR, Fawzi WW. Prevalence and predictors of adolescent linear growth and stunting across the urban–rural gradient in eastern Ethiopia. Trop Med Int Health. 2020 Jan;25(1):101–10. doi: 10.1111/tmi.13341 31710743

[95] Tamrat A, Yeshaw Y, Dadi AF. Stunting and Its Associated Factors among Early Adolescent School Girls of Gondar Town, Northwest Ethiopia: A School-Based Cross-Sectional Study. F Faramawi M, editor. BioMed Res Int. 2020 Oct 23;2020:1–6. 10.1155/2020/8850074 PMC7604580 33163537

[96] Andargie M, Gebremariam K, Hailu T, Addisu A, Zereabruk K. Magnitude of Overweight and Obesity and Associated Factors Among Public and Private Secondary School Adolescent Students in Mekelle City, Tigray Region, Ethiopia, 2019: Comparative Cross-Sectional Study. Diabetes Metab Syndr Obes Targets Ther. 2021 Mar; Volume 14:901–15. doi: 10.2147/DMSO.S262480 33688225 PMC7936680

[97] Worku M, Gizaw Z, Kassahun Belew A, Wagnew A, Hunegnaw MT. Prevalence and Associated Factors of Overweight and Obesity among High School Adolescents in Bahir Dar City, Northwest, Ethiopia: A Cross-Sectional Study. Ardern CI, editor. J Obes. 2021 Mar 9;2021:1–8. doi: 10.1155/2021/8846723 33777450 PMC7969120

[98] Kebede D, Prasad RPCJ, Asres DT, Aragaw H, Worku E. Prevalence and associated factors of stunting and thinness among adolescent students in Finote Selam Town, Northwest Ethiopia. J Health Popul Nutr. 2021 Dec;40(1):44. doi: 10.1186/s41043-021-00269-4 34663482 PMC8524843

[99] Alemu TG, Muhye AB, Ayele AD. Under nutrition and associated factors among adolescent girls attending school in the rural and urban districts of Debark, Northwest Ethiopia: A community-based comparative cross-sectional study. Bhargava M, editor. PLOS ONE. 2021 Aug 16;16(8):e0254166. 34398878 10.1371/journal.pone.0254166 PMC8366968

[100] Handiso YH, Belachew T, Abuye C, Workicho A, Baye K. Undernutrition and its determinants among adolescent girls in low land area of Southern Ethiopia. Darteh EKM, editor. PLOS ONE. 2021 Jan 12;16(1):e0240677. doi: 10.1371/journal.pone.0240677 33434212 PMC7802945

[101] Hadush G, Seid O, Wuneh AG. Assessment of nutritional status and associated factors among adolescent girls in Afar, Northeastern Ethiopia: a cross-sectional study. J Health Popul Nutr. 2021 Dec;40(1):2. doi: 10.1186/s41043-021-00227-0 33622414 PMC7903644

[102] Ashebir Kebede W, Yimer Ayele B. Magnitude of Stunting and Associated Factors among Adolescent Students in Legehida District, Northeast Ethiopia. Gumpricht E, editor. J Nutr Metab. 2021 Oct 15;2021:1–7. doi: 10.1155/2021/2467883 34691778 PMC8536425

[103] Mulu Birru G, Eshete Tadesse S, Hassen Abate K, Mekonnen TC, Genetu Chane M. Malnutrition in School-Going Adolescents in Dessie Town, South Wollo, Ethiopia. Huerta JM, editor. J Nutr Metab. 2021 Jan 7;2021:1–8. doi: 10.1155/2021/4898970 33520306 PMC7817239

[104] Kedir S, Hassen K, Melaku Y, Jemal M. Determinants of overweight and/or obesity among school adolescents in Butajira Town, Southern Ethiopia. A case-control study. Vall-llosera Camps M, editor. PLOS ONE. 2022 Jun 28;17(6):e0270628. doi: 10.1371/journal.pone.0270628 35763506 PMC9239474

[105] Tafasa SM, Tura MR, Mulu E, Begna Z. Undernutrition and its associated factors among school adolescent girls in Abuna Gindeberet district, Central Ethiopia: a cross-sectional study. BMC Nutr. 2022 Aug 24;8(1):87. doi: 10.1186/s40795-022-00587-8 36002840 PMC9400317

[106] Belay M, Oumer A, Abdureshid N, Ale A. Overnutrition and Associated Factors Among High School Adolescents in Mid COVID-19 Pandemic in Ethiopia: Neglected Public Health Concern. Adolesc Health Med Ther. 2022 Jan; Volume 13:1–14. doi: 10.2147/AHMT.S349189 35082546 PMC8784252

[107] Frongillo EA, Nanama S. Development and Validation of an Experience-Based Measure of Household Food Insecurity within and across Seasons in Northern Burkina Faso. J Nutr. 2006 May 1;136(5):1409S–1419S. doi: 10.1093/jn/136.5.1409S 16614438

[108] World Health Organizations: Adolescent Nutrition: A review of the situation in selected South-East Asian countries: Available at: https://www.who.int/nutrition/publications/schoolagechildren/SEA_NUT_163/en/.

[109] Caleyachetty R, Thomas GN, Kengne AP, Echouffo-Tcheugui JB, Schilsky S, Khodabocus J, et al. The double burden of malnutrition among adolescents: analysis of data from the Global School-Based Student Health and Health Behavior in School-Aged Children surveys in 57 low- and middle-income countries. Am J Clin Nutr. 2018 Aug 1;108(2):414–24. doi: 10.1093/ajcn/nqy105 29947727

[110] Abera M, Hardy-Johnson P, Abdissa A, Workicho A, Ali R, Weller S, et al. Social, economic and cultural influences on adolescent nutrition and physical activity in Jimma, Ethiopia: perspectives from adolescents and their caregivers. Public Health Nutr. 2020 Jul 30;1–9. doi: 10.1017/S1368980020001664 32727633 PMC10195353

[111] Seleshe S, Jo C, Lee M. Meat Consumption Culture in Ethiopia. Korean J Food Sci Anim Resour. 2014;34(1):7–13. doi: 10.5851/kosfa.2014.34.1.7 26760739 PMC4597829

[112] Baye TG. Poverty, peasantry and agriculture in Ethiopia. Ann Agrar Sci. 2017 Sep;15(3):420–30.

[113] Food and Agriculture Organization of the United Nations (FAO). United Nations Decade of Action on Nutrition 2016–2025. Asia and the Pacific Symposium on Sustainable Food Systems for Healthy Diets and Improved Nutrition–Accelerating Nutrition, Symposium report. 2018. Rome, 64 pp.

[114] United Nations System Standing Committee on Nutrition (UNSCN). By 2030, end all forms of malnutrition and leave no one behind. Discussion Paper, 2017.

[115] National Adolescent and Youth Health Strategy (2016–2020): Federal Democratic Republic Of Ethiopia, Ministry Of Health, 2016.

[116] Guideline: implementing effective actions for improving adolescent nutrition. Geneva: World Health Organization; 2018. Licence: CC BY-NC-SA 3.0 IGO.

[117] Burt MR. Reasons to invest in adolescents. J Adolesc Health. 2002 Dec;31(6):136–52. doi: 10.1016/s1054-139x(02)00486-x 12470910

[118] Nutrition in adolescence–Issues and Challenges for the Health Sector Issues in Adolescent Health and Development: World Health Organization 2005; ISBN 92 4 159366 0.

