# Correction: A systematic review and meta-analysis of adolescent nutrition in Ethiopia: Transforming adolescent lives through nutrition (TALENT) initiative

**DOI:** 10.1371/journal.pone.0347523

**Published:** 2026-04-16

**Authors:** Mubarek Abera, Abdulhalik Workicho, Melkamu Berhane, Desta Hiko, Rahma Ali, Beakal Zinab, Abraham Haileamlak, Caroline Fall

The corresponding author, Mubarek Abera, email address is incorrect. The correct email address is: mubarek.abera@ju.edu.et.

In Fig 7, the red box currently labeled “Body” should be corrected to “Boy”. Please see the correct Fig 7 here.

**Fig 7:**
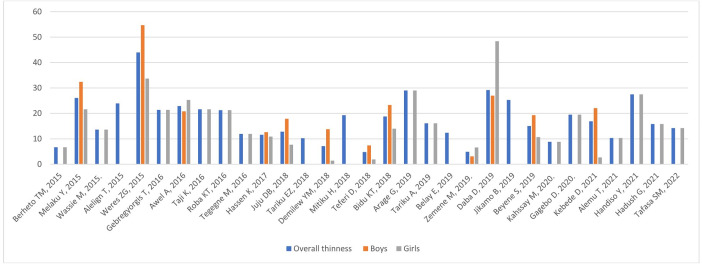
Trends in adolescent thinness by sex in Ethiopia over the last 6 years.
