# Supplementary material for: A systematic review and meta-analysis of adolescent nutrition in Ethiopia: Transforming adolescent lives through nutrition (TALENT) initiative
Source: PLoS One. 2023 Apr 6;18(4):e0280784. doi: 10.1371/journal.pone.0280784 (PMC10079135; doi:10.1371/journal.pone.0280784)
Supplement: S1 Checklist — (DOCX) [file pone.0280784.s001.docx]

| S.No | Articles | Was the sample frame appropriate to address the target population? | Were study participants sampled in an appropriate way? | Was the sample size adequate? | Were the study subjects and the setting described in detail? | Was the data analysis conducted with sufficient coverage of the identified sample? | Were valid methods used for the identification of the condition? | Was the condition measured in a standard, reliable way for all participants? | Was there appropriate statistical analysis? | Was the response rate adequate, and if not, was the low response rate managed appropriately |
| --- | --- | --- | --- | --- | --- | --- | --- | --- | --- | --- |
|  | Alemayehu T 2010 | Yes | Yes | Yes | Yes | Yes | Yes | Yes | Yes | Yes |
|  | Herrador Z., 2014 | No | Yes | Yes | Yes | Yes | Yes | Yes | Yes | Yes |
|  | Gebreyohannes Y, 2014. | Yes | Yes | Yes | Yes | Yes | Yes | Yes | Yes | Yes |
|  | Berheto TM 2015 | Yes | Yes | Yes | Yes | Yes | Yes | Yes | Yes | Yes |
|  | Melaku Y. 2015 | Yes | Yes | Yes | Yes | Yes | Yes | Yes | Yes | Yes |
|  | Assefa H, 2015 | Yes | Yes | Yes | Yes | Yes | Yes | Yes | Yes | Yes |
|  | Wassie M, 2015. | Yes | Yes | Yes | Yes | No | Yes | Yes | Yes | Yes |
|  | Alelign T 2015 | No | Yes | Yes | Yes | Yes | Yes | Yes | Yes | Yes |
|  | Weres ZG 2015 | Yes | Yes | Yes | Yes | Yes | Yes | Yes | Yes | Yes |
|  | Gebregyorgis T. 2016 | Yes | Yes | Yes | Yes | Yes | Yes | Yes | Yes | Yes |
|  | Awel A 2016 | Yes | Yes | Yes | Yes | Yes | Yes | Yes | Yes | Yes |
|  | Taji K. 2016 | Yes | Yes | Yes | Yes | Yes | Yes | Yes | Yes | Yes |
|  | Roba KT 2016 | Yes | Yes | Yes | Yes | Yes | Yes | Yes | Yes | Yes |
|  | Tegegne M 2016 | Yes | Yes | Yes | Yes | Yes | Yes | Yes | Yes | Yes |
|  | Shegaze M 2016 | Yes | Yes | Yes | Yes | Yes | Yes | Yes | Yes | Yes |
|  | Gali N 2017 | Yes | Yes | Yes | Yes | Yes | Yes | Yes | Yes | Yes |
|  | Hassen K. 2017 | Yes | Yes | Yes | Yes | Yes | Yes | Yes | Yes | Yes |
|  | Juju DB., 2018 | Yes | Yes | Yes | Yes | Yes | Yes | Yes | Yes | Yes |
|  | Birru S, 2018. | Yes | Yes | Yes | Yes | Yes | Yes | Yes | Yes | Yes |
|  | Mekonnen T, 2018. | No | Yes | Yes | Yes | Yes | Yes | Yes | Yes | Yes |
|  | Tariku EZ. 2018 | No | Yes | Yes | Yes | Yes | Yes | Yes | Yes | Yes |
|  | Moges T, 2018. | Yes | Yes | Yes | Yes | Yes | Yes | Yes | Yes | Yes |
|  | Demilew YM 2018 | Yes | Yes | Yes | Yes | Yes | Yes | Yes | Yes | Yes |
|  | Mitiku H 2018 | No | Yes | Yes | Yes | Yes | Yes | Yes | Yes | Yes |
|  | Teferi D, 2018 | Yes | Yes | Yes | Yes | Yes | Yes | Yes | Yes | Yes |
|  | Girmay A, 2018 | Yes | Yes | Yes | Yes | Yes | Yes | Yes | Yes | Yes |
|  | Bidu KT 2018 | Yes | Yes | Yes | Yes | Yes | Yes | Yes | Yes | Yes |
|  | Arage G, 2019 | Yes | Yes | Yes | Yes | Yes | Yes | Yes | Yes | Yes |
|  | Tariku A.. 2019. | Yes | Yes | Yes | Yes | Yes | Yes | Yes | Yes | Yes |
|  | Belay E, 2019 | No | Yes | Yes | Yes | Yes | Yes | Yes | Yes | Yes |
|  | Zemene M, 2019. | Yes | Yes | Yes | Yes | Yes | Yes | Yes | Yes | Yes |
|  | Wolde T. 2019 | Yes | Yes | Yes | Yes | Yes | Yes | Yes | Yes | Yes |
|  | Daba D 2019 | Yes | Yes | Yes | Yes | Yes | Yes | Yes | Yes | Yes |
|  | Jikamo B, 2019 | Yes | Yes | Yes | Yes | Yes | Yes | Yes | Yes | Yes |
|  | Beyene S 2019 | Yes | Yes | Yes | Yes | Yes | Yes | Yes | Yes | Yes |
|  | Berhe K, 2020 | Yes | Yes | Yes | Yes | Yes | Yes | Yes | Yes | Yes |
|  | Kahssay M. 2020. | Yes | Yes | Yes | Yes | Yes | Yes | Yes | Yes | Yes |
|  | Gagebo D. 2020. | Yes | Yes | Yes | Yes | Yes | Yes | Yes | Yes | Yes |
|  | Taklual W, 2020 | Yes | Yes | Yes | Yes | Yes | Yes | Yes | Yes | Yes |
|  | Tamrat A, 2020 | Yes | Yes | Yes | Yes | Yes | Yes | Yes | Yes | Yes |
|  | Irenso A, 2020 | Yes | Yes | Yes | Yes | Yes | Yes | Yes | Yes | Yes |
|  | Andargie M, 2020 | Yes | Yes | Yes | Yes | Yes | Yes | Yes | Yes | Yes |
|  | Sisay B, 2020 | Yes | Yes | Yes | Yes | Yes | Yes | Yes | Yes | Yes |
|  | Birru GM, 2021 | Yes | Yes | Yes | Yes | Yes | Yes | Yes | Yes | Yes |
|  | Kebede WA, 2021 | Yes | Yes | Yes | Yes | Yes | Yes | Yes | Yes | Yes |
|  | Worku M, 2021 | Yes | Yes | Yes | Yes | Yes | Yes | Yes | Yes | Yes |
|  | Kebede D, 2021 | Yes | Yes | Yes | Yes | Yes | Yes | Yes | Yes | Yes |
|  | Alemu T, 2021 | Yes | Yes | Yes | Yes | Yes | Yes | Yes | Yes | Yes |
|  | Handiso Y, 2021 | Yes | Yes | Yes | Yes | Yes | Yes | Yes | Yes | Yes |
|  | Hadush G, 2021 | Yes | Yes | Yes | Yes | Yes | Yes | Yes | Yes | Yes |
|  | Kedir S, 2022 | Yes | Yes | Yes | Yes | Yes | Yes | Yes | Yes | Yes |
|  | Tafasa, S.M. 2022 | Yes | Yes | Yes | Yes | Yes | Yes | Yes | Yes | Yes |
|  | Belay M, 2022 | Yes | Yes | Yes | Yes | Yes | Yes | Yes | Yes | Yes |
|  | Desalegn D, 2014 | Yes | Yes | Yes | Yes | Yes | Yes | Yes | Yes | Yes |
|  | Wakao T, *2015* | Yes | Yes | Yes | Yes | Yes | Yes | Yes | Yes | Yes |
|  | DHS report, 2016 | Yes | Yes | Yes | Yes | Yes | Yes | Yes | Yes | Yes |
|  | Ministry of Health, 2016 | Yes | Yes | Yes | Yes | Yes | Yes | Yes | Yes | Yes |
|  | Teji K, 2016 | Yes | Yes | Yes | Yes | Yes | Yes | Yes | Yes | Yes |
|  | Getaneh Z, 2017 | Yes | Yes | Yes | Yes | Yes | Yes | Yes | Yes | Yes |
|  | Workie S, 2017 | Yes | Yes | Yes | Yes | Yes | Yes | Yes | Yes | Yes |
|  | Gonete K, 2017 | Yes | Yes | Yes | Yes | Yes | Yes | Yes | Yes | Yes |
|  | Wakayo T, 2018 | Yes | Yes | Yes | Yes | Yes | Yes | Yes | Yes | Yes |
|  | Seyoum Y, 2019 | Yes | Yes | Yes | Yes | Yes | Yes | Yes | Yes | Yes |
|  | Mengistu G 2019 | Yes | Yes | Yes | Yes | Yes | Yes | Yes | Yes | Yes |
|  | Demelash S 2019 | Yes | Yes | Yes | Yes | Yes | Yes | Yes | Yes | Yes |
|  | Gebreyesus SH 2019 | Yes | Yes | Yes | Yes | Yes | Yes | Yes | Yes | Yes |
|  | Regasa RT, 2019 | Yes | Yes | Yes | Yes | Yes | Yes | Yes | Yes | Yes |
|  | Gebremichael G, 2020 | Yes | Yes | Yes | Yes | Yes | Yes | Yes | Yes | Yes |
|  | Mulugeta A, 2015 | Yes | Yes | Yes | Yes | Yes | Yes | Yes | Yes | Yes |
|  | Tamiru D, 2016 | Yes | Yes | Yes | Yes | Yes | Yes | Yes | Yes | Yes |
|  | Melaku Y 2017 | Yes | Yes | Yes | Yes | Yes | Yes | Yes | Yes | Yes |
|  | Birru SM, 2018 | Yes | Yes | Yes | Yes | Yes | Yes | Yes | Yes | Yes |
|  | Hadley C, 2008 | Yes | Yes | Yes | Yes | Yes | Yes | Yes | Yes | Yes |
|  | Belachew T, 2012 | Yes | Yes | Yes | Yes | Yes | Yes | Yes | Yes | Yes |
|  | Belachew T, 2013 | Yes | Yes | Yes | Yes | Yes | Yes | Yes | Yes | Yes |
|  | Belachew T, 2013 | Yes | Yes | Yes | Yes | Yes | Yes | Yes | Yes | Yes |
|  | Mulusew G, 2017 | Yes | Yes | Yes | Yes | Yes | Yes | Yes | Yes | Yes |
|  | Gizaw G, 2018 | Yes | Yes | Yes | Yes | Yes | Yes | Yes | Yes | Yes |
