# Supplementary material for: A systematic review and meta-analysis of adolescent nutrition in Ethiopia: Transforming adolescent lives through nutrition (TALENT) initiative
Source: PLoS One. 2023 Apr 6;18(4):e0280784. doi: 10.1371/journal.pone.0280784 (PMC10079135; doi:10.1371/journal.pone.0280784)
Supplement: Attachment — Submitted filename: Response to Review Feedback.docx [file pone.0280784.s005.docx]

**Response to Reviewers Feedback: Journal PLOS ONE**

The authors are very thankful for the editor’s and reviewer’s feedback to improve the quality of the manuscript further. We have accepted and incorporated the comments in the main text, tables, figures and additional files. Here the point by point response in presented.

**Feedback given**

1. We are concerned that the literature review was completed in 2020. Systematic and scoping reviews should provide an up-to-date overview of the research topic covered. Since the literature search was completed over a year ago, we are concerned that this study does not meet this requirement.

**Response:** comment accepted and the search is extended to cover July 20, 2022. Due to this additional search additional articles are included in to the review and update has been done accordingly.

2. We would also require that you replace the standard PRISMA checklist with a PRISMA-ScR checklist (available at <https://www.equator-network.org/wp-content/uploads/2018/09/PRISMA-ScR-Fillable-Checklist-1.docx>), and that you complete all the relevant items.

**Response**: Comment accepted and the PRISMA-ScR checklist is now used.
